# Tissue-targeted inorganic pyrophosphate hydrolysis in a *fugu5* mutant reveals that excess inorganic pyrophosphate triggers developmental defects in a cell-autonomous manner

**DOI:** 10.3389/fpls.2022.945225

**Published:** 2022-08-04

**Authors:** Shizuka Gunji, Kensuke Kawade, Hiromitsu Tabeta, Gorou Horiguchi, Akira Oikawa, Mariko Asaoka, Masami Yokota Hirai, Hirokazu Tsukaya, Ali Ferjani

**Affiliations:** ^1^Department of Biology, Tokyo Gakugei University, Koganei, Tokyo, Japan; ^2^United Graduate School of Education, Tokyo Gakugei University, Tokyo, Japan; ^3^National Institute for Basic Biology, Okazaki, Aichi, Japan; ^4^Department of Basic Biology, School of Life Sciences, Graduate University for Advanced Studies (SOKENDAI), Okazaki, Aichi, Japan; ^5^RIKEN Center for Sustainable Resource Science, Yokohama, Japan; ^6^Department of Life Science, College of Science, Rikkyo University, Tokyo, Japan; ^7^Research Center for Life Science, College of Science, Rikkyo University, Tokyo, Japan; ^8^Faculty of Agriculture, Yamagata University, Tsuruoka, Japan; ^9^Department of Applied Biosciences, Graduate School of Bioagricultural Science, Nagoya University, Nagoya, Japan; ^10^Department of Biological Sciences, Graduate School of Science, The University of Tokyo, Tokyo, Japan

**Keywords:** *fugu5* mutant, leaf morphogenesis, metabolism, pyrophosphate homeostasis, pyrophosphatase, cell-autonomous regulation

## Abstract

Excess PPi triggers developmental defects in a cell-autonomous manner. The level of inorganic pyrophosphate (PPi) must be tightly regulated in all kingdoms for the proper execution of cellular functions. In plants, the vacuolar proton pyrophosphatase (H^+^-PPase) has a pivotal role in PPi homeostasis. We previously demonstrated that the excess cytosolic PPi in the H^+^-PPase loss-of-function *fugu5* mutant inhibits gluconeogenesis from seed storage lipids, arrests cell division in cotyledonary palisade tissue, and triggers a compensated cell enlargement (CCE). Moreover, PPi alters pavement cell (PC) shape, stomatal patterning, and functioning, supporting specific yet broad inhibitory effects of PPi on leaf morphogenesis. Whereas these developmental defects were totally rescued by the expression of the yeast soluble pyrophosphatase IPP1, sucrose supply alone canceled CCE in the palisade tissue but not the epidermal developmental defects. Hence, we postulated that the latter are likely triggered by excess PPi rather than a sucrose deficit. To formally test this hypothesis, we adopted a spatiotemporal approach by constructing and analyzing *fugu5-1 PDF1_*pro*_::IPP1*, *fugu5-1 CLV1_*pro*_::IPP1*, and *fugu5-1 ICL_*pro*_::IPP1*, whereby PPi was removed specifically from the epidermis, palisade tissue cells, or during the 4 days following seed imbibition, respectively. It is important to note that whereas PC defects in *fugu5-1 PDF1_*pro*_::IPP1* were completely recovered, those in *fugu5-1 CLV1_*pro*_::IPP1* were not. In addition, phenotypic analyses of *fugu5-1 ICL_*pro*_::IPP1* lines demonstrated that the immediate removal of PPi after seed imbibition markedly improved overall plant growth, abolished CCE, but only partially restored the epidermal developmental defects. Next, the impact of spatial and temporal removal of PPi was investigated by capillary electrophoresis time-of-flight mass spectrometry (CE-TOF MS). Our analysis revealed that the metabolic profiles are differentially affected among all the above transgenic lines, and consistent with an axial role of central metabolism of gluconeogenesis in CCE. Taken together, this study provides a conceptual framework to unveil metabolic fluctuations within leaf tissues with high spatio–temporal resolution. Finally, our findings suggest that excess PPi exerts its inhibitory effect *in planta* in the early stages of seedling establishment in a tissue- and cell-autonomous manner.

## Introduction

Pyrophosphate (PPi) is ubiquitously released within living cells as a byproduct of nearly 200 metabolic reactions, such as DNA, RNA, protein, and saccharide polymerization (Stitt, 1998; Maeshima, 2000; Heinonen, 2001; Ferjani et al., 2014a,b). In plant cells, PPi is used as an intracellular energy donor by several enzymes, such as the tonoplastic PPi-dependent proton pump (H^+^-PPase; Rea and Poole, 1985, 1993; Rea et al., 1992; Sarafian et al., 1992; Maeshima, 2000; Drozdowicz and Rea, 2001), UDP-glucose pyrophosphorylase (UGPase, EC 2.7.7.9), fructose-6-phosphate 1-phosphotransferase (PFP, EC 2.7.1.90; Stitt, 1990; Dennis et al., 1997), and pyruvate phosphate dikinase (PPDK, EC 2.7.9.1; Parsley and Hibberd, 2006; Eastmond et al., 2015). Controlling the PPi level is essential to sustaining cellular activity. The loss of function of soluble PPase (sPPase) in several organisms, such as *Escherichia coli* (Chen et al., 1990), *Saccharomyces cerevisiae* (Lundin et al., 1991), and *Caenorhabditis elegans* (Ko et al., 2007), leads to severe growth arrest and/or cell death due to excess PPi. Such severe phenotype or lethality has for decades hampered corroboration of the effects of a perturbed PPi level *in planta*.

We reported that excess PPi in the H^+^-PPase loss-of-function *fugu5* mutant severely compromises gluconeogenesis from seed storage lipids and inhibits a hypocotyl elongation in etiolated seedlings (Ferjani et al., 2011, 2014a, 2018), arrests cell division in cotyledonary palisade tissue, and triggers a compensated cell enlargement (CCE; Tsukaya, 2002, 2008; Horiguchi et al., 2005, 2006a,b; Ferjani et al., 2007, 2008, 2010, 2011, 2013a,b; Amano et al., 2015; Katano et al., 2016; Takahashi et al., 2017; Tabeta et al., 2021; Nakayama et al., 2022). It is important to note that these phenotypes were recovered by exogenous sucrose (Suc) supply or specific removal of PPi from the *fugu5* background by the yeast cytosolic PPase IPP1 under the control of the vacuolar H^+^-pyrophosphatase *AVP1*/*FUGU5* promoter in *fugu5-1 AVP1_*pro*_::IPP1* transgenic lines (Ferjani et al., 2011, 2014a,b; Asaoka et al., 2019; Gunji et al., 2020). Consistently, CE-TOF MS analyses of the major metabolites characteristic of gluconeogenesis from seed storage lipids identify UGPase as the major target of the inhibitory effects of excess PPi *in planta* (Ferjani et al., 2018). Moreover, UGPase, a ubiquitous enzyme found in bacteria, animals, and plants catalyzes the production of UDP-glucose (UDP-Glc), the major glycosyl donor for Suc synthesis, as well as cellulose and callose formation and is the precursor of other nucleotide sugars (Kleczkowski, 1994; Gibeaut, 2000; Winter and Huber, 2000; Johansson et al., 2002).

The *Arabidopsis thaliana* (hereafter, Arabidopsis) genome encodes five cytosolic sPPases (*AtPPa1* to *AtPPa5*), and one isoform (*AtPPa6*) was reported to be targeted to the chloroplast stroma (Schulze et al., 2004; Öztürk et al., 2014; Gutiérrez-Luna et al., 2016). Moreover, sPPases and H^+^-PPase cooperatively regulate the PPi level: H^+^-PPase is essential for maintaining adequate PPi content, and the cytosolic AtPPa isozymes, in particular, AtPPa1, prevent its increase to a toxic level (Ferjani et al., 2014a; Segami et al., 2018).

In plants, H^+^-PPase mediates the hydrolysis of PPi and the acidification of the vacuole (Rea and Poole, 1985; Rea et al., 1992; Sarafian et al., 1992; Maeshima, 2000; Drozdowicz and Rea, 2001). It has long been thought that H^+^-PPase is important as an H^+^-pump; however, analyses of *fugu5 AVP1_*pro*_::IPP1* provided evidence that the hydrolysis of inhibitory PPi, rather than vacuolar acidification, is the major contribution of this enzyme during the establishment of Arabidopsis seedlings (Ferjani et al., 2011; Segami et al., 2018). However, until recently, the key questions concerning the effects of excess PPi on leaf tissue other than the palisade and other cell types have gone unanswered.

Examination of the outermost cotyledon layer in *fugu5* revealed an increase in stomatal density (i.e., the number of stomata per unit area) in violation of the one-cell-spacing rule, a defect in stomata closure (Asaoka et al., 2019), and showed that pavement cells (PCs) exhibit defective puzzle-cell formation (Gunji et al., 2020). It is important to note that specific removal of PPi in *fugu5-1 AVP1_*pro*_::IPP1* transgenic lines restored these epidermal phenotypic aberrations in the *fugu5* background (Gunji et al., 2020). Furthermore, in *fugu5 GC1_*pro*_::IPP1* transgenic lines, which exclusively express IPP1 in guard cells, stomatal closure recovered, which suggests a role for H^+^-PPase in stomatal function (Asaoka et al., 2019). It is surprising that PCs in mutants with defects in gluconeogenesis (*pck1-2*; Eastmond et al., 2000) or the glyoxylate cycle (*icl-2* and *mls-2*; Cornah et al., 2004; Penfield et al., 2004, respectively) showed no phenotypic change, which indicates that reduced Suc production from seed storage lipids is not the cause of *fugu5* epidermal developmental defects (Gunji et al., 2020). The subsequent live imaging revealed that cortical microtubules (MTs) in PCs exhibited a reduced velocity and were slightly fragmented and sparse in *fugu5* compared to the wildtype (WT; Gunji et al., 2020). Consistently, the addition of PPi *in vitro* leads to a dose-dependent delay in tubulin polymerization, thus, supporting a link between PPi and MT dynamics (Gunji et al., 2020). Taken together, these findings suggest that whereas CCE is triggered by decreased Suc production, epidermal defects are likely triggered by an overaccumulation of PPi. Hence, whereas PPi homeostasis is a prerequisite for proper leaf organogenesis, excess PPi has differential effects on the growth and development of palisade tissue cells and epidermal cells.

Here, we used a spatiotemporal approach to unpack the molecular process underlying the inhibitory effects of PPi on the key leaf tissues and at the key developmental stages. More specifically, we constructed *PDF1_*pro*_::IPP1*, *CLV1_*pro*_::IPP1*, and *ICL_*pro*_::IPP1* lines in which PPi was removed, on the *fugu5-1* background, from the epidermis, palisade tissue cells, or during the 4 days following seed imbibition, respectively. The phenotypic and metabolomics approaches showed that excess PPi *in planta* has its inhibitory effect in the early stages of seedling establishment and in a tissue-autonomous manner.

## Materials and methods

### Plant materials and growth conditions

The WT used in this study was *A. thaliana* Colombia-0 (Col-0), and mutants and all transgenic plants were on the Col-0 background. Also, *fugu5-1* was isolated and characterized as a loss-of-function mutant of the vacuolar type H^+^-PPase (Horiguchi et al., 2006b; Ferjani et al., 2007, 2011). In addition, the two previously described independent transgenic lines expressing yeast sPPase IPP1 on the *fugu5-1* mutant background (*AVP1_*pro*_::IPP1*#8-3 and #17-3) were used (Ferjani et al., 2011). The seeds were sown on rockwool (Nippon Rockwool), watered daily with 0.5 g L^–1^ Hyponex solution, and grown under a 16-h light and 8-h dark cycle with white light from fluorescent lamps at approximately 50 μmol m^–2^ s^–1^ and 22°C. The sterilized seeds were sown on Suc-free Murashige and Skoog (MS) medium (Wako Pure Chemical) or MS medium with 2% (w/v) Suc, where indicated. 0.1% (w/v) 2-(N-morpholino) ethanesulfonic acid (MES) was added, then pH was adjusted to 5.8 with KOH, and solidified with 0.2–0.5% (w/v) gellan gum (Murashige and Skoog, 1962) to determine the effects of medium composition on phenotype. The seeds were sown on MS plates and stored at 4°C in the dark for 3 days as cold treatment. After the cold treatment, the seedlings were grown in the light (as above) or in the dark for the indicated periods of time.

### Generation of *PDF1_*pro*_::IPP1* and *CLV1_*pro*_::IPP1* transgenic plants

About 1.5 kbp of the upstream region of the *PDF1* gene and about 2.0 kbp of the upstream region of the *CLV1* gene that had been cloned into the pDONR P4-P1R were used (Kawade et al., 2013). The coding region of *IPP1* from *S. cerevisiae* was fused into pDONR201 (Ferjani et al., 2011), and the vectors were subjected to the LR reaction with the R4 gateway binary vector R4pGWB501 (Nakagawa et al., 2008). The resultant final constructs were used to transform *fugu5-1* mutant plants by the floral–dip method (Clough and Bent, 1998). Several independent T_3_ homozygous lines expressing the *IPP1* gene under the control of *PDF1*_*pro*_ (*PDF1_*pro*_::IPP1*#6-7, *PDF1_*pro*_::IPP1*#9-1, and *PDF1_*pro*_::IPP1*#20-5 lines) or *CLV1*_*pro*_ (*CLV1_*pro*_::IPP1*#8-3, *CLV1_*pro*_::IPP1*#10-2, and *CLV1_*pro*_::IPP1*#12-3 lines) from a single T-DNA insertion locus on the *fugu5-1* background were used for analyses.

### Obtaining *PDF1_*pro*_::GUS* and *CLV1_*pro*_::GUS*

On the *an3-4* background, *PDF1_*pro*_::GUS* and *CLV1_*pro*_::GUS* were described elsewhere (Kawade et al., 2013). To obtain *PDF1_*pro*_::GUS* and *CLV1_*pro*_::GUS* on the *fugu5-1* background, we crossed *fugu5-1* with the above lines and used the resultant F_3_ generation to select double homozygous lines based on hygromycin resistance and *fugu5-1* genotyping, as described previously (Takahashi et al., 2017).

### Generation of *ICL_*pro*_::GUS* and *ICL_*pro*_::IPP1* transgenic plants

For *ICL* promoter *GUS* construction, the *ICL* promoter was amplified by PCR with the pICL-F: 5′-TATATGTTTGAAGCTCATCCACGAGCTAAGCAAGT-3′ and pICL-R: 5′-GGACTGACCTACCCGGGCTTTAACTTTTA TAAATTGG-3′ primer set. The binary vector pSMAB704 was digested with *Sma*I and *Hind*III to remove the 35S promoter. The PCR-amplified *ICL* promoter fragments were cloned into the *Sma*I and *Hind*III sites of pSMAB704 by infusion (TaKaRa). The resulting vectors were treated with *Sma*I and *Sac*I to remove the *GUS* cDNA coding region. Using the IPP1-F: 5′-TAAAAGTTAAAGCCCATGACCTACACTACCAGACA-3′ and IPP1-R: 5′-GATCGGGGAAATTCGTTAAACAGAACCGG AGATGA-3′ primer set, we cloned amplified *IPP1* into the *ICL_*pro*_::GUS Sma*I and *Sac*I sites by infusion (TaKaRa). The resultant final construct was used to transform *fugu5-1* mutant plants by the floral–dip method (Clough and Bent, 1998). Several independent T_3_ homozygous lines expressing *IPP1* under the *ICL* promoter were identified on the *fugu5-1* background. The *ICL* promoter lines *ICL_*pro*_::IPP1*#9-2, *ICL_*pro*_::IPP1*#11-2, *ICL_*pro*_::IPP1*#13-1, and *ICL_*pro*_::IPP1*#24-1 were used in the further experiments.

### The GUS Histochemical Staining

The seedlings were treated with 90% (v/v) acetone on ice. For GUS staining, the samples were incubated in staining solution [sterile distilled water, 750 μg/ml X-Gluc (5-bromo-4-chloro-3-indolyl-β-D-glucuronide), 100-mM sodium phosphate buffer (pH 7.0), 3-mM potassium ferrocyanide, 10-mM ethylenediaminetetraacetic acid buffer (pH 8.0), and [0.1% (v/v) Triton X-100] overnight at 37°C in a desiccator. The GUS-stained samples were washed once with sterile Milli-Q, cleared with chloral solution, and observed under a light stereomicroscope (Leica Microsystems).

### Microscopy and phenotypic analysis

The photographs of gross plant morphology at 10 days after seed sowing (DAS) and 12 DAS were taken with a stereoscopic microscope (M165FC; Leica Microsystems) connected to a CCD camera (DFC300FX; Leica Microsystems) and a digital camera (D5000 Nikkor lens AF-S Micro Nikkor 60 mm; Nikon). The gross plant morphology at 25 DAS was photographed with a digital camera (D5000 Nikkor lens AF-S Micro Nikkor 60 mm; Nikon). After sampling, the leaves were fixed in formalin/acetic acid/alcohol [FAA; 4% (v/v) formalin, 5% (v/v) acetic acid, and 50% (v/v) ethanol] and cleared with chloral solution (200-g chloral hydrate, 20-g glycerol, and 50-ml deionized water) to measure the leaf area and cell number, as described previously (Tsuge et al., 1996). The whole leaves were observed with a stereomicroscope equipped with a CCD camera. The leaf palisade tissue cells were observed and photographed under a light microscope (DM-2500; Leica Microsystems) equipped with Nomarski differential interference contrast optics and a CCD camera. The cell size was determined as the mean palisade cell area of 20 palisade cells per leaf on a paravermal view (Ferjani et al., 2011).

### Observation and quantitative analysis of epidermal cells

For scanning electron microscopy (SEM), cotyledons were dissected from the plants at the indicated growth stages. The samples were fixed overnight in FAA at room temperature. The fixed specimens were dehydrated in an ethanol series [50, 60, 70, 80, 90, 95, 99.5, and 100% (v/v); 60 min per step] and stored overnight in 100% (v/v) ethanol at room temperature. The ethanol was replaced with 3-methylbutyl acetate, and the samples were dried in a critical-point dryer (JCPD-5; JEOL), sputter-coated with gold–palladium using an anion sputter (JFC-1100; JEOL), and examined under an S-3400N scanning electron microscope (Hitachi) as described previously (Maeda et al., 2014). The SEM images of the adaxial side of cotyledons were used to quantify PC complexity. The area and perimeter of individual PCs (*n* = 20–30 cells from one cotyledon; a total of six cotyledons) were measured with ImageJ (v.1.63). We quantified their complexity by calculating the undulation index (UI; Thomas et al., 2003) using the following equation (Kürschner, 1997):


$$\text{UI}=\frac{Ce}{2\pi \sqrt{Ae/\pi }},$$

where UI (dimensionless) is the undulation index, *C* (μm) is the cell perimeter, and A (μm^2^) is the cell area. Note that an increased UI indicates an increased PC complexity and *vice versa*.

Stomatal index (SI) is the percentage of the number of stomata to the total number of epidermal cells. Stomatal index was calculated using the following equation:


SI=Stx 100E+S,

where St is the number of stomata per unit area, and E is the number of epidermal cells within the same unit area.

### Metabolite profiling by capillary electrophoresis time-of-flight mass spectrometry (CE-TOF MS)

Three-day-old etiolated seedlings were immediately frozen in liquid nitrogen after sampling and stored at –80°C prior to CE-TOF MS analysis. The CE-TOF MS analysis was performed as described previously (Ferjani et al., 2018); further details are presented elsewhere (Oikawa et al., 2011a,b). The raw CE-TOF MS data were converted, and the peaks were automatically identified, aligned, and annotated with our in-house software Masterhands (Sugimoto et al., 2010).

### Quantitative analysis of total triacylglycerol (TAG)

We measured the quantities of seed lipid reserves in dry seeds and in 1-, 2-, 3-, and 4-day-old etiolated seedlings by determining total TAG using a Triglyceride E-Test Assay Kit (Wako Pure Chemical). Either 20 dry seeds or 20 seedlings were homogenized with a mortar and pestle in 100 μl sterile distilled water. The homogenates were mixed with 0.75 ml of the reaction buffer provided in the kit as described previously (Arai et al., 2008; Ferjani et al., 2011). The sample TAG concentration was determined according to the manufacturer’s protocol. The lengths of etiolated seedlings were determined as described previously (Ferjani et al., 2011).

### Quantification of Suc

A total of 100 etiolated seedlings at 3 days after induction of seed germination (DAI) were collected in a tube in liquid nitrogen and lyophilized. The samples were extracted with a bead shocker in a 2 ml tube with 5 mm zirconia beads and 80% MeOH for 2 min at 1000 rpm (Shake Master NEO; Biomedical Sciences). The extracted solutions were centrifuged at 104 g for 1 min, and 150-μl centrifuged solution and 10-μl 2 mg/L (UL-^13^C_6_*^glc^*)-Suc (Omicron Biochemicals, South Bend, IN, United States) were dispensed into a 1.5-ml tube. After the solution was dried with a centrifuge evaporator (Speed vac; Thermo), 100-μl Mox reagent (2% methoxyamine in pyridine; Thermo) was added to the 1.5-ml tube, and the metabolites were methoxylated at 30°C and 1,200 rpm for approximately 6 h with a thermo-shaker (BSR-MSC100; Biomedical Sciences). After methoxylation, 50-μl 1% v/v trimethylchlorosilane (TMS; Thermo) was added to the 1.5-ml tube. For TMS derivatization, the mixture was incubated for 30 min at 1,200 rpm at 37°C as above. Finally, 50 μl of the derivatized samples were dispensed into vials for GC-QqQ-MS analysis (AOC-5000 Plus with GCMS-TQ8040; Shimadzu) as described elsewhere (Tabeta et al., 2021). The raw data were collected, and GC-MS peak areas were calculated with GCMS software (Shimadzu). Suc content was quantified per etiolated seedling based on an internal standard.

### Quantification of plant fresh weight (FW)

To measure FW, we used 12-day-old seedlings and 25-day-old plants grown on rockwool. Five 12-day-old seedlings were pooled, and their weight was measured (*n* = 10 biological replicates) with a precision balance (MS204S; Mettler Toledo). For 25-day-old plants, FW was measured (*n* = 20) with an electronic scale (TE1502S; Sartorius).

### Statistical analysis

The data were subjected to statistical analyses by one-way ANOVA with Tukey’s honestly significant difference (HSD) test (Kaleida Graph, v.4.1.1). The metabolome statistical analyses were conducted with MetaboAnalyst 4.0 and 5.0 (Chong et al., 2019; Pang et al., 2021^1^). The PCA plots were generated in MetaboAnalyst 5.0 (Pang et al., 2021). The selection of metabolites for the Venn diagram was based on Tukey’s HSD test in MetaboAnalyst 4.0 (Supplementary Tables 2, 4), and the Venn diagram was generated with the R package VennDiagram (Chen, 2018; v.1.6.20; R v.3.6.3; R Core Team, 2020). Hierarchical cluster analyses (HCA) and heat maps were created with the R package pheatmap (Kolde, 2019; v.1.0.12; R v.3.5.1; R Core Team, 2018). The calculation of autoscaling sample data for HCA was performed in MetaboAnalyst 4.0 (Chong et al., 2019).

## Results

### Tissue-specific expression of IPP1 affect morphological and cellular phenotypes in the *fugu5* background

**FIGURE 1:**
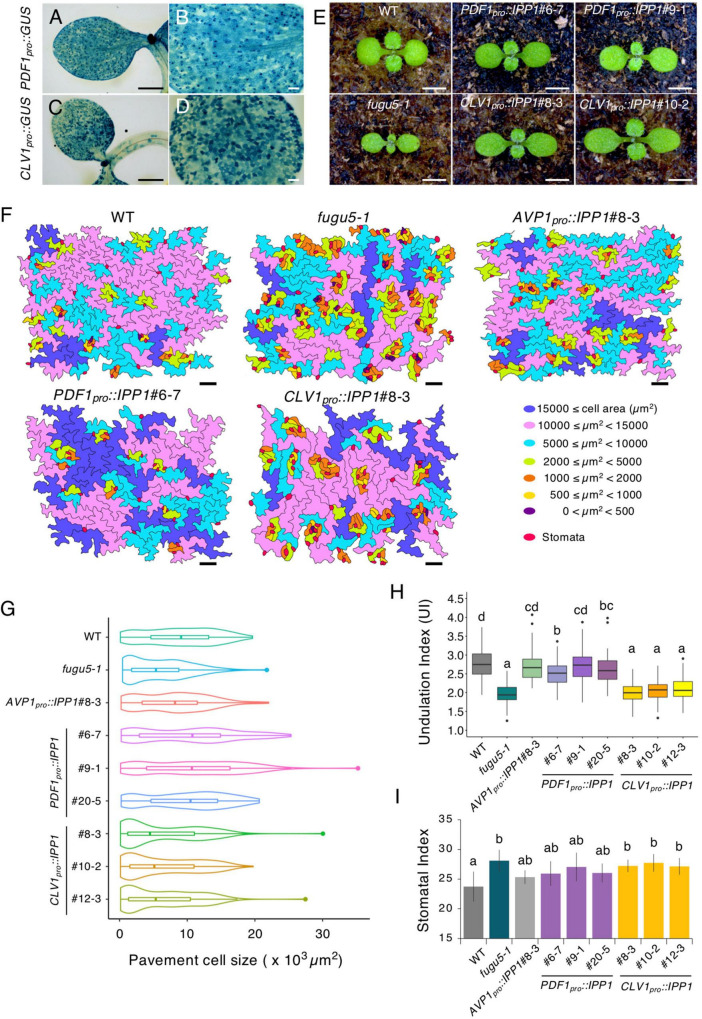
Phenotypes of transgenic plants expressing IPP1 in a tissue-specific manner. **(A–D)** The GUS staining of 5-day-old seedlings of *PDF1_*pro*_::GUS* and *CLV1_*pro*_::GUS* grown on MS medium. **(B,D)** are magnified images of **(A,C)**, respectively. Scale bar = 500 μm **(A,C)** and 100 μm **(B,D)**. **(E)** Gross morphology of representative transgenic lines grown on rockwool for 10 DAS. Scale bar = 2 mm. **(F)** Pavement cells (PCs) of the adaxial side on representative scanning electron microscopy (SEM) images were traced and color-coded based on cell size. Scale bar = 100 μm. **(G)** Violin plots of the size distribution of PCs, excluding stomata (*n* = 200 cells from four independent cotyledons). **(H)** Box-plot represents undulation index (UI) of PCs (*n* = 125 PCs from six cotyledons) at 25 DAS. The boxes of lower and upper are the first and third quantiles, the line in the boxes is the median and whiskers are maximum and minimum values. **(I)** The stomatal index was determined as the number of stomata per 100 PCs. Results are means ± SD (*n* = 6 cotyledons). Each character represents a significant difference at *p <* 0.05 (Tukey’s HSD test). DAS, days after seed sowing.

It is reported that *AVP1*_*pro*_ is active throughout the plant body (Segami et al., 2014), and hence it is unsuitable for evaluating the inhibitory effects of PPi in the palisade or the epidermis or at a given developmental stage. Whereas CCE in *fugu5* is triggered by a lack of Suc, the developmental defects in the *fugu5* epidermis are assumed to be triggered by excess PPi. To formally test this hypothesis, we examined the effects of tissue-specific hydrolysis of PPi. We used the *PROTODERMAL FACTOR 1* (*PDF1*_*pro*_) and *CLAVATA1* (*CLV1*_*pro*_) promoters because they are active in the epidermis and in the subepidermal layer of palisade tissue, respectively (Abe et al., 2001; Kawade et al., 2013; Asaoka et al., 2021; Figures 1A–D). Moreover, *PDF1_*pro*_::IPP1* and *CLV1_*pro*_::IPP1* transgenic lines were constructed on the *fugu5-1* background and characterized in detail.

Although *fugu5* displayed a delayed post-germinative growth, expression of *PDF1_*pro*_::IPP1* and *CLV1_*pro*_::IPP1* on the *fugu5-1* background substantially improved overall plant growth (Figure 1E; Supplementary Figures 1, 2A,B). Whereas *PDF1_*pro*_::IPP1* lines displayed round cotyledons, *CLV1_*pro*_::IPP1* cotyledons were oblong, mimicking the *fugu5-1* single mutant (Figure 1E; Supplementary Figure 2C).

Next, to assess the epidermis phenotype, we conducted SEM observation of the cotyledon adaxial side. The SEM images revealed that the clusters of stomata had formed in *fugu5-1*, which proved to be consistent with our previous report (Asaoka et al., 2019; Figure 1F; Supplementary Figure 3). Moreover, PCs in *fugu5-1* and *CLV1_*pro*_::IPP1* lines were smaller compared to the WT, *PDF1_*pro*_::IPP1*, and *AVP1_*pro*_::IPP1* lines (Figures 1F,G). Consistently, PCs in *fugu5-1* exhibited simpler shapes than the WT (Gunji et al., 2020; Figures 1F,H). The undulation index (UI) values revealed that the WT and *PDF1_*pro*_::IPP1* were comparable (Figure 1H). By contrast, UI values in the *CLV1_*pro*_::IPP1* independent lines were similar to those of *fugu5-1* (Figure 1H). Although the stomatal index (SI) value was higher in *fugu5-1* and *CLV1_*pro*_::IPP1*, it was partially restored in *PDF1_*pro*_::IPP1* (Figure 1I).

### Compensation is only partially affected upon tissue-specific expression of IPP1

After seed imbibition, TAG of the oil bodies, which is the major seed storage lipid in Arabidopsis, is converted into Suc *via* a sequence of metabolic processes (Graham, 2008). In one study of *fugu5*, the failure to hydrolyze PPi led to a ∼2.5-fold increase in its level compared to the WT (Ferjani et al., 2011). The excess PPi compromises the gluconeogenic enzyme UGPase, reducing levels of UDP-Glc and Suc (Ferjani et al., 2011, 2018). By contrast, oil bodies accumulate in the epidermal and mesophyll tissue of embryonic cotyledons within mature seeds of Arabidopsis. Hence, what are the effects of tissue-specific expression of IPP1 on gluconeogenesis and thus CCE?

**FIGURE 2:**
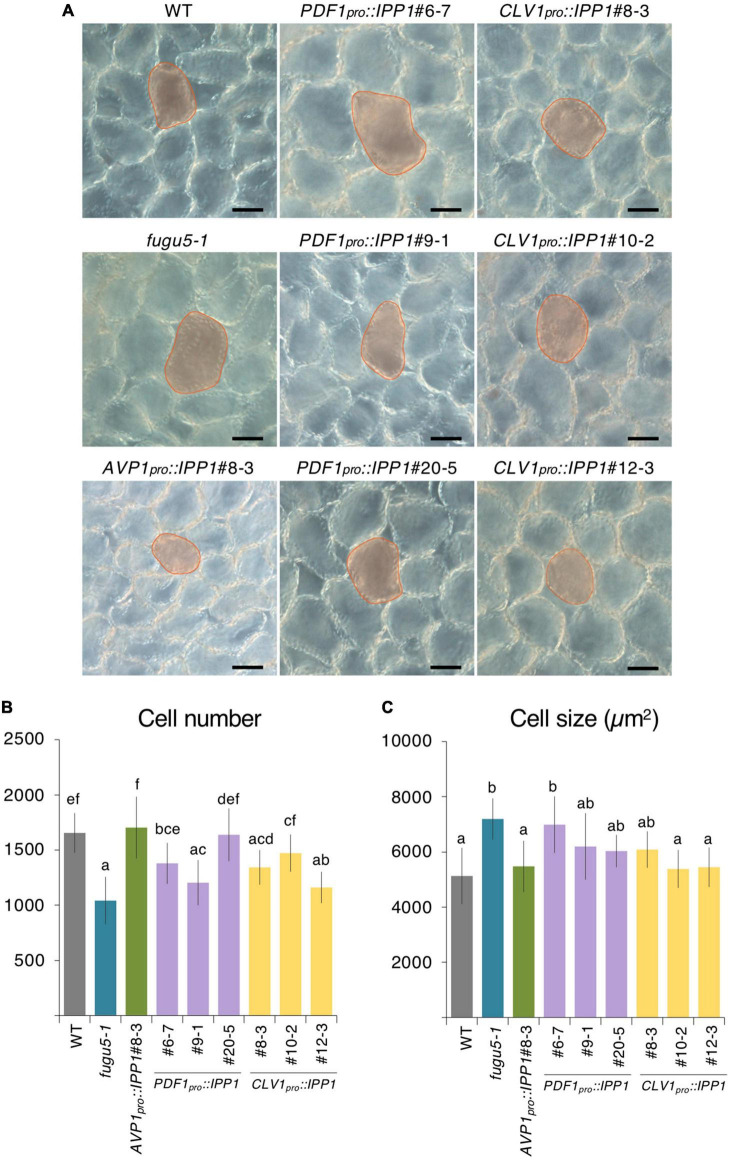
Palisade cellular phenotypes of mature cotyledons from the *PDF1_*pro*_::IPP1* and *CLV1_*pro*_::IPP1* transgenic lines. **(A)** Micrographs of palisade tissue cells from cleared cotyledons of the wildtype (WT), *fugu5-1*, *AVP1_*pro*_::IPP1*#8-3, *PDF1_*pro*_::IPP1*#6-7, *PDF1_*pro*_::IPP1*#9-1, *PDF1_*pro*_::IPP1*#20-5, *CLV1_*pro*_::IPP1*#8-3, *CLV1_*pro*_::IPP1*#10-2, and *CLV1_*pro*_::IPP1*#12-3 lines at 25 DAS. Red contour and shadow highlight representative cell size in each genotype. Scale bar = 50 μm. **(B,C)** Numbers of subepidermal palisade tissue cells **(B)** and their average sizes **(C)**. Data are means ± SD (*n* ≥ 9 cotyledons). Each character represents a significant difference at *p <* 0.05 (Tukey’s HSD test). DAS, days after seed sowing.

To address this question, we performed the quantitative and comparative analyses of the cell number and size of palisade tissue in the WT, *fugu5-1*, *AVP1_*pro*_::IPP1*#8-3, three independent *PDF1_*pro*_::IPP1* lines, and three independent *CLV1_*pro*_::IPP1* lines (Figure 2). Consistent with our previous reports, *fugu5-1* cell number and size in cotyledons were totally recovered in *AVP1_*pro*_::IPP1*#8-3 (Ferjani et al., 2011; Figures 2B,C). By contrast, the targeted expression of IPP1 in the epidermis or palisade tissue had a small effect on cell number and size (Figures 2B,C). Given the ubiquitous expression of *AVP1*_*pro*_ (Segami et al., 2018) and the tissue-specific expression of *PDF1*_*pro*_ and *CLV1*_*pro*_, and the fact that compensation in *fugu5-1* is triggered exclusively by defective TAG-to-Suc conversion, it is plausible that in *PDF1_*pro*_::IPP1* and *CLV1_*pro*_::IPP1* Suc production did not fully recover.

To corroborate this hypothesis, we crossed *PDF1_*pro*_::IPP1*#6-7 with *CLV1_*pro*_::IPP1*#8-3 and evaluated the cellular phenotypes in F_1_ progeny heterozygous for both transgenes (Supplementary Figure 4). We were interested to find that CCE was abolished in the double transgenic heterozygous lines (Supplementary Figures 4A–D).

### Impact of tissue-specific expression of IPP1 on hypocotyl elongation, triacylglycerol (TAG) mobilization and gluconeogenesis

**FIGURE 3:**
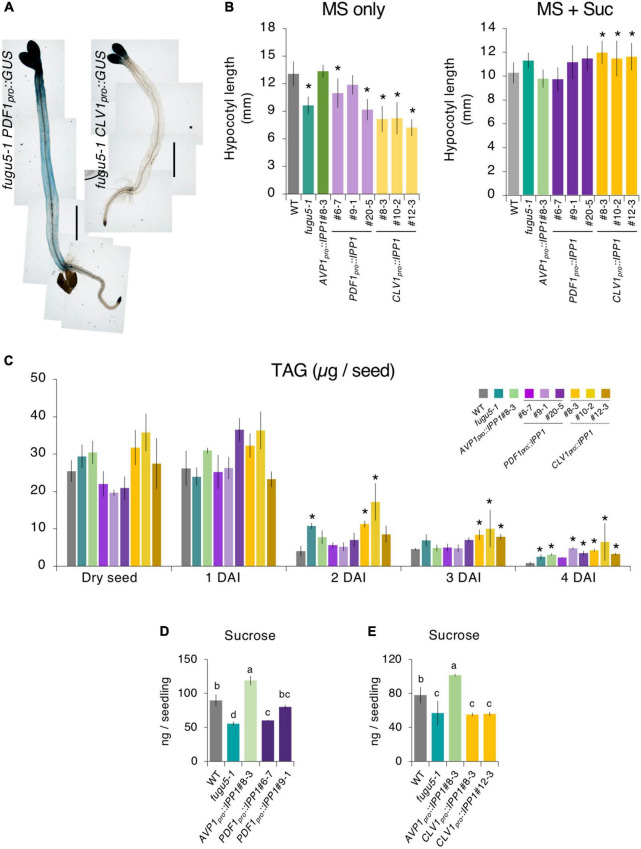
Etiolated seedling elongation and triacylglycerol (TAG) mobilization in transgenic plants expressing IPP1 in a tissue-specific manner. **(A)** Micrographs of GUS staining of etiolated seedlings of the *fugu5-1 PDF1_*pro*_::GUS* and *fugu5-1 CLV1_*pro*_::GUS* lines grown on MS medium in darkness for 4 DAI. Scale bar = 1 mm. **(B)** Length of hypocotyls of the wildtype (WT), *fugu5-1*, *AVP1_*pro*_::IPP1*#8-3, *PDF1_*pro*_::IPP1*, and *CLV1_*pro*_::IPP1* lines grown on MS only and MS + Suc medium in the dark for 4 DAI. Data are means ± SD (*n* = 21). *Significant difference at *p* < 0.05 compared to the WT (Tukey’s HSD test). **(C)** Mobilization of seed lipid reserves during post-germinative growth. The TAG content was quantified with 20 dry seeds or 20 etiolated seedlings at 1, 2, 3, and 4 DAI. Data are means ± SD (*n* = 3 independent experiments). *Significant difference at *p* < 0.05 compared to the WT (Tukey’s HSD test). **(D,E)** Sucrose levels determined by GC-MS/MS of 100 etiolated seedlings at 3 DAI. Data are means ± SD (*n* = 3 independent experiments). Each character represents a significant difference at *p <* 0.05 (Tukey’s HSD test). DAI, days after induction of seed germination.

In darkness and in the absence of exogenous Suc, a hypocotyl elongation relies on TAG-to-Suc conversion as the sole source of energy (Eastmond et al., 2000; Cornah et al., 2004; Penfield et al., 2004; Katano et al., 2016; Takahashi et al., 2017). We examined expression of *PDF1*_*pro*_ and *CLV1*_*pro*_ using *PDF1_*pro*_::GUS* and *CLV1_*pro*_::GUS* reporter lines. GUS staining of etiolated seedlings at 4 DAI revealed that both *PDF1_*pro*_::GUS* and *CLV1_*pro*_::GUS* were strongly expressed in cotyledons (Figure 3A). By contrast, whereas *PDF1_*pro*_::GUS* was evenly expressed in the hypocotyl, *CLV1_*pro*_::GUS* was not detected in this organ (Figure 3A). Therefore, we hypothesized that the removal of PPi was affected in *PDF1_*pro*_::IPP1* and *CLV1_*pro*_::IPP1*, altering Suc availability and thus affecting etiolated seedling elongation. To evaluate this hypothesis, we sowed seeds on MS only or MS + 2% Suc medium and allowed them to grow in darkness for 4 DAI. Quantification of the length of the hypocotyl grown on MS only revealed that although *PDF1_*pro*_::IPP1* tended to be longer than *fugu5-1*, the length of *CLV1_*pro*_::IPP1* etiolated seedlings did not recover (Figure 3B). Next, the amount of TAG in dry seeds and in 1, 2, 3, and 4 DAI etiolated seedlings was quantified as reported previously (Arai et al., 2008; Ferjani et al., 2011; Takahashi et al., 2017). It is interesting that the TAG content in dry seeds and their degradation during etiolated seedling establishment were similar in all genotypes except *CLV1_*pro*_::IPP1*, in which TAG degradation was slightly delayed, in particular, at 2 and 3 DAI (Figure 3C).

Finally, to evaluate the impact of tissue-specific expression of IPP1 on gluconeogenesis, we quantified Suc content by GC-MS/MS (Tabeta et al., 2021). The Suc level in *PDF1_*pro*_::IPP1* tended to increase (Figure 3D), whereas that in *CLV1_*pro*_::IPP1* was indistinguishable from *fugu5-1* (Figure 3E).

### Removal of inorganic pyrophosphate (PPi) immediately after seed imbibition promotes plant growth

The pleiotropic phenotype of the *fugu5* cotyledon is triggered by excess PPi produced following metabolic reactivation in the embryo upon seed imbibition. However, the post-germinative developmental stage susceptible to excess PPi is unclear.

Because *IPP1* was expressed constitutively in *AVP1_*pro*_::IPP1*, *PDF1_*pro*_::IPP1*, and *CLV1_*pro*_::IPP1*, we assessed the contribution of PPi hydrolysis. To this end, the promoter of *ISOCITRATE LYASE* (*ICL*), a key enzyme in the peroxisomal glyoxylate cycle, was used because *ICL* transcription increased rapidly after seed imbibition, remained high for 3 days, and was negligible after 4 days (Eastmond et al., 2000). To investigate the inhibitory effects of PPi after seed imbibition on the *fugu5-1* background, we generated *ICL_*pro*_::GUS* and *ICL_*pro*_::IPP1* transgenic lines on the *fugu5-1* background.

**FIGURE 4:**
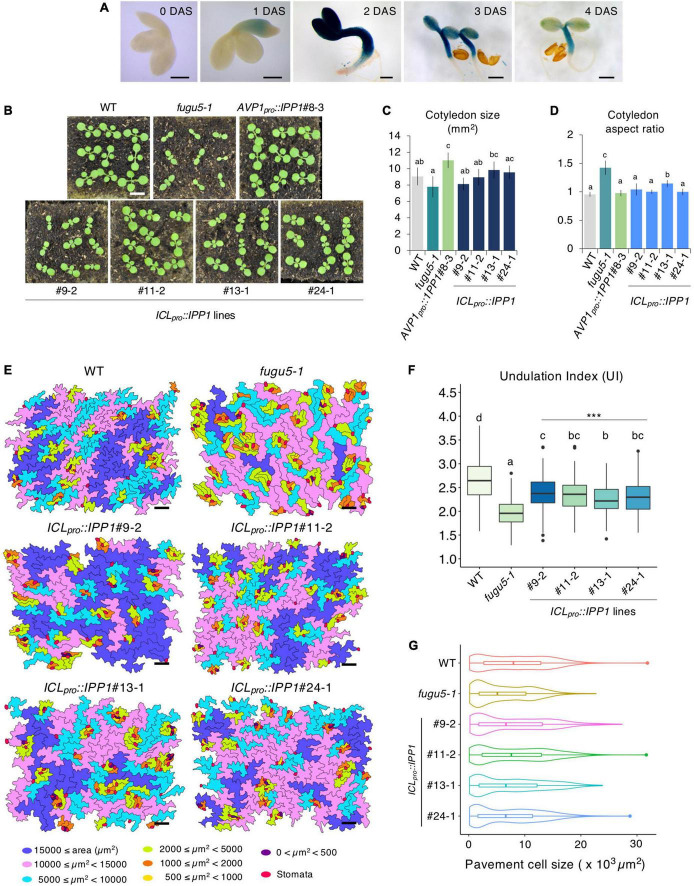
Effect of developmental stage-specific removal of inorganic pyrophosphate (PPi) on plant development. **(A)** Time course of GUS expression in *fugu5-1 ICL_*pro*_::GUS*. Scale bar = 200 μm (at 0-, 1-, 2 DAS); Scale bar = 500 μm (at 3 and 4 DAS). **(B)** Gross morphology of the wildtype (WT), *fugu5-1*, *AVP1_*pro*_::IPP1*#8-3, and *ICL_*pro*_::IPP1* lines. Photographs of 10-day-old plants grown on rockwool. Scale bar = 5 mm. **(C)** Cotyledon size and **(D)** aspect ratio of six cotyledons at 25 DAS. Data are means ± SD. Each character represents a significant difference at *p* < 0.05 (Tukey’s HSD test). **(E)** PCs in representative scanning electron microscopy (SEM) images of the WT, *fugu5-1*, *ICL_*pro*_::IPP1*#9-2, *ICL_*pro*_::IPP1*#11-2, *ICL_*pro*_::IPP1*#13-1, and *ICL_*pro*_::IPP1*#24-1 lines were traced and color-coded based on their size. Scale bar = 100 μm. **(F)** Box-plot represents undulation index (UI) of PCs (*n* = 180 PCs from six cotyledons) at 25 DAS. The boxes of lower and upper are the first and third quantiles, the line in the boxes is the median and whiskers are maximum and minimum values. Each character represents a significant difference at *p* < 0.05. ***Significant difference compared to *fugu5-1* at *p* < 0.001 (Tukey’s HSD test). **(G)** Violin plots of the size distribution of PCs, excluding stomata (*n* = 480 cells from six cotyledons). DAS, days after seed sowing.

*ICL_*pro*_::GUS* expression in the cotyledon increased gradually following seed imbibition, peaked at 2 DAS, and decreased markedly at 3–4 DAS (Figure 4A). The post-germinative growth of *ICL_*pro*_::IPP1* seedlings at 10 (Figure 4B), 14, and 23 (Supplementary Figure 5A) DAS was enhanced compared to *fugu5-1* and cotyledon size and aspect ratio were almost indistinguishable from the WT (Figures 4C,D). Indeed, *ICL_*pro*_::IPP1* growth was markedly improved compared to *fugu5-1* in terms of FW, which increased 150–260% at 12 DAS and 140–180% at 25 DAS (Supplementary Figure 5B,C). Therefore, temporal removal of PPi upon seed imbibition and germination significantly promotes seedling growth at early stages, which enhances the subsequent growth of the plant.

### Removal of inorganic pyrophosphate (PPi) immediately after seed imbibition partially rescues the epidermal developmental defects

To corroborate how the removal of PPi within 3,4 days of germination promotes plant growth, we quantified the shape and size of cotyledon PCs in the *ICL_*pro*_::IPP1* transgenic line (Figures 4E–G; Supplementary Figure 6). The UI values of PCs in *ICL_*pro*_::IPP1* were intermediate between the WT and *fugu5-1* single mutant (Figure 4F). PCs in *fugu5-1* are reportedly significantly smaller compared to the WT, in which cells less than 2,000 μm^2^ account for more than or 30% of cotyledon PCs (Gunji et al., 2020). The number of cells less than 2,000 μm^2^ was significantly decreased in *ICL_*pro*_::IPP1*, and the proportion of cells > 15,000 μm^2^ was increased (Figures 4E,G). Together, these findings indicate that the expression of IPP1 to a confined window of a few days following the seed germination is efficient but not enough to fully restore the PC jigsaw puzzle-like pattern that is generated by interdigitation of the cell wall during cotyledon development.

### Triacylglycerol (TAG)-to-Suc conversion restores cell number and cancels compensated cell enlargement (CCE) in *ICL_*pro*_::IPP1*

**FIGURE 5:**
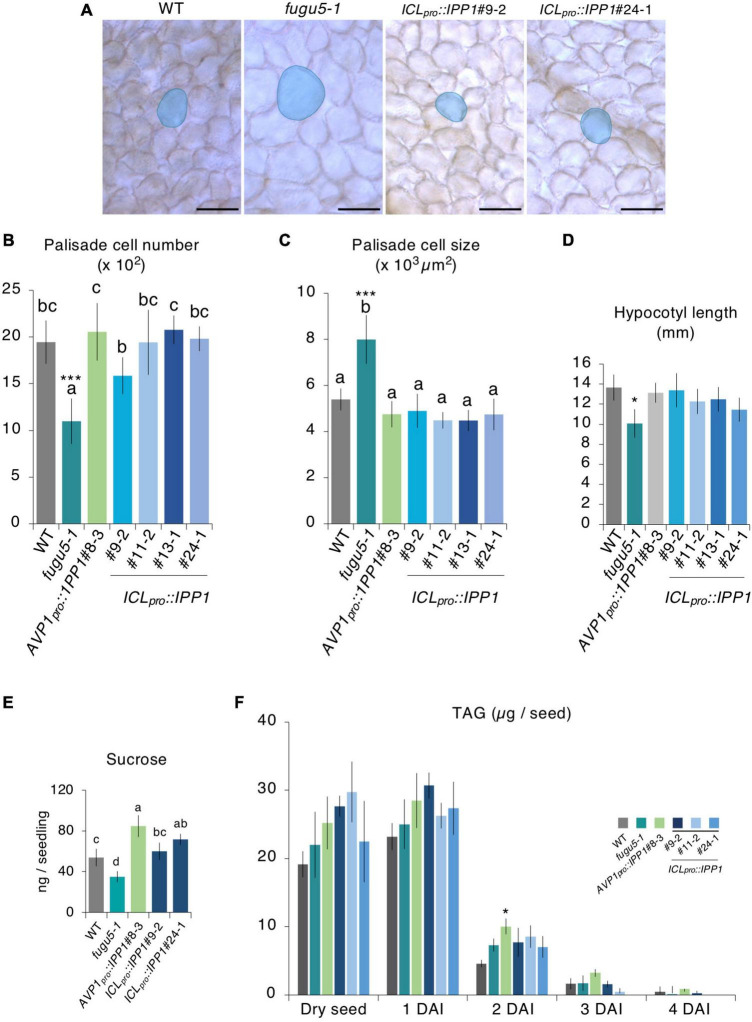
Effect of developmental stage-specific removal of inorganic pyrophosphate (PPi) on cotyledon cellular phenotype and etiolated seedling elongation. **(A)** Micrographs of cleared cotyledon palisade tissue cells from the wildtype (WT), *fugu5-1*, *ICL_*pro*_::IPP1*#9-2, and *ICL_*pro*_::IPP1*#24-1 at 25 DAS. Blue contour and shadow highlight representative cell size in each genotype. Scale bar = 100 μm. **(B)** Numbers and **(C)** sizes of cotyledon subepidermal palisade tissue cells; means ± SD (*n* = 6 cotyledons). Each character represents a significant difference at *p* < 0.05. ***Significant difference at *p* < 0.0001 compared to the WT (Tukey’s HSD test). **(D)** Hypocotyl length at 4 DAI; means ± SD (*n* = 21 seedlings). *Significant difference at *p* < 0.01 compared to the WT (Tukey’s HSD test). **(E)** Sucrose levels of 100 etiolated seedlings at 3 DAI by GC-MS/MS. Data are means ± SD (*n* = 4 independent experiments). Each character represents a significant difference at *p <* 0.05 (Tukey’s HSD test). **(F)** Time course of the mobilization of triacylglycerol (TAG) during post-germinative growth. The TAG content was quantified with 20 dry seeds or 20 etiolated seedlings at 1, 2, 3, and 4 DAI. Data are means ± SD (*n* = 3 independent experiments). *Significant difference at *p* < 0.05 compared to the WT. DAS, days after seed sowing, DAI, days after induction of seed germination.

Excess PPi affects both the epidermis by altering PC shape (Gunji et al., 2020) and the palisade tissue cells in which CCE is triggered (Bertoni, 2011; Ferjani et al., 2011). The inhibitory mechanisms are different and independent for the two layers. To evaluate the phenotype of palisade tissue cells, namely, CCE, we quantified cell number and size in *ICL_*pro*_::IPP1*. It is interesting that the cell number in the palisade tissue of the transgenic line recovered to the WT level, and CCE did not occur (Figures 5A–C). Provided that CCE in *fugu5-1* is triggered by a partial failure of TAG mobilization, transient expression of IPP1 under the control of *ICL*_*pro*_ is likely necessary and sufficient to provide the required amount of carbon for energy production to reactivate cell cycling in palisade tissue immediately after seed imbibition (Bertoni, 2011; Ferjani et al., 2011).

The seedlings kept in darkness adopt a skotomorphogenic developmental program, in which allocation of resources is typically directed toward a hypocotyl elongation at the expense of cotyledon and root development (Josse and Halliday, 2008). Indeed, the lack of Suc in *fugu5-1* compromises a hypocotyl elongation in darkness, resulting in ∼30% shorter hypocotyls compared to the WT (Ferjani et al., 2011; Katano et al., 2016). To evaluate the effects of *ICL_*pro*_::IPP1* expression on the skotomorphogenic developmental program, we quantified the length of 4 DAI etiolated seedlings. Hypocotyl length in *ICL_*pro*_::IPP1* significantly recovered to the WT level in the absence of exogenous Suc (Figure 5D). Indeed, *ICL_*pro*_::IPP1* produced as much Suc as the WT (Figure 5E). The TAG mobilization was not affected in *ICL_*pro*_::IPP1*, in agreement with our finding for the *fugu5-1* single mutant (Ferjani et al., 2011; Katano et al., 2016; Tabeta et al., 2021; Figure 5F). Taken together, these results indicate that PPi removal at the early stages of seed germination is critical for an efficient TAG-to-Suc conversion and re-establishment of normal palisade cell number and size.

### Tissue-specific removal of inorganic pyrophosphate (PPi) in *PDF1_*pro*_:IPP1* and *CLV1_*pro*_::IPP1* affects metabolic profiles

The expression of *PDF1_*pro*_::IPP1* and *CLV1_*pro*_::IPP1* on the *fugu5-1* background had distinct effects on epidermis and palisade tissue morphogenesis. Specifically, although the removal of PPi from the epidermis by *PDF1_*pro*_::IPP1* was sufficient to restore the PC phenotype, *CLV1_*pro*_::IPP1* expression had only a partial effect (Figures 1F–I). Hence, the effects of PPi differ by leaf tissue, and we postulated that it would also have different impacts on the metabolic profiles of the transgenic lines. Because the conversion of glucose 1-phosphate (G1P) to UDP-Glc is inhibited by excess PPi on the *fugu5* background (Ferjani et al., 2018), we used CE-TOF MS to evaluate metabolic changes in *PDF1_*pro*_::IPP1* and *CLV1_*pro*_::IPP1* compared to *fugu5-1*.

**FIGURE 6:**
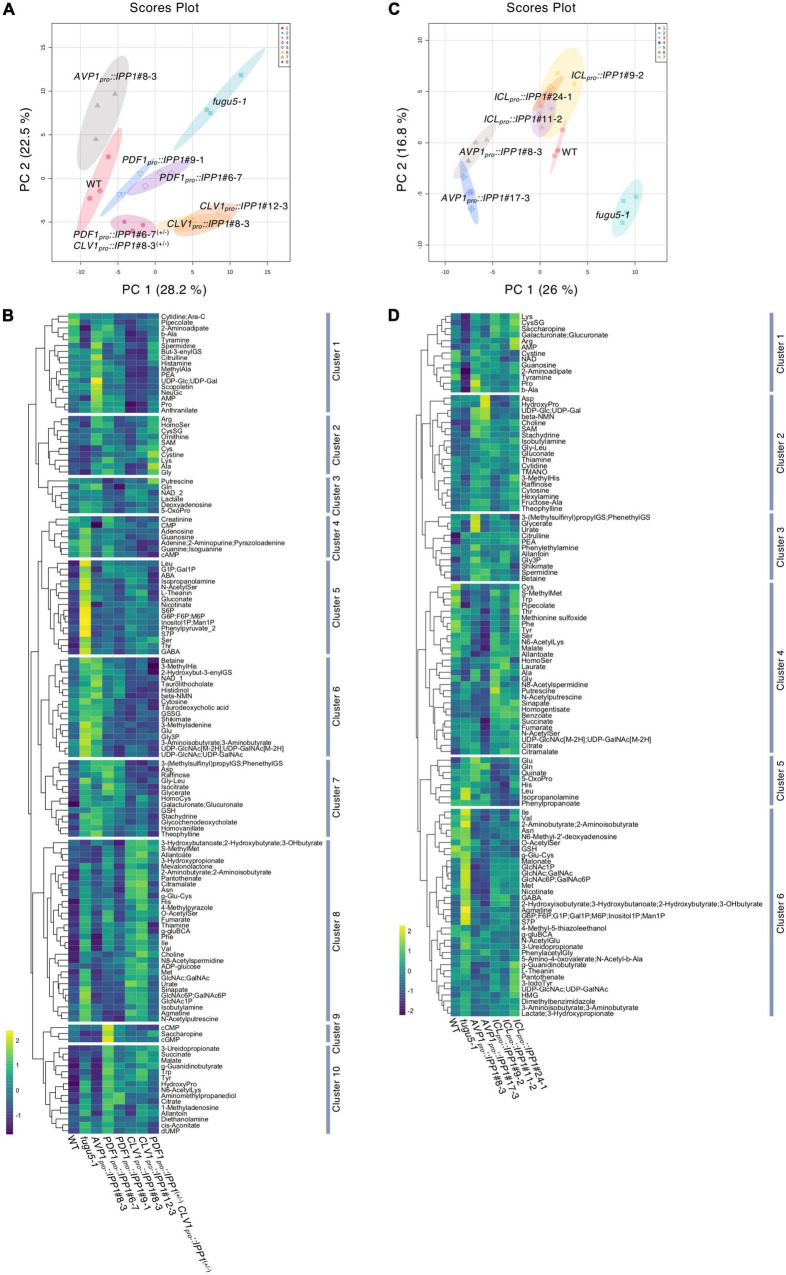
Effect of spatiotemporal expression of IPP1 on the *fugu5* background on the metabolic profiles of etiolated seedlings determined by capillary electrophoresis time-of-flight mass spectrometry (CE-TOF MS). The MetaboAnalyst (v.5.0) website was used to normalize the data and perform principal component analysis (PCA). The normalization procedures consisted of mean-centering and division by the standard deviation of each variable. **(A)** The PCA plot based on three independent experiments involving the wildtype (WT), *fugu5-1*, *AVP1_*pro*_::IPP1*#8-3, *PDF1_*pro*_::IPP1*#6-7, *PDF1_*pro*_::IPP1*#9-1, *CLV1_*pro*_::IPP1*#8-3, *CLV1_*pro*_::IPP1*#12-3, and *PDF1_*pro*_::IPP1*#6-7^(+/–)^ *CLV1_*pro*_::IPP1*#8-3^(+/–)^ lines. **(B)** Hierarchical clustering heat map of the relative abundances of metabolites. Scale indicates the color code relative to normalized metabolite abundance. **(C)** PCA score plot from four independent experiments involving the WT, *fugu5-1*, *AVP1_*pro*_::IPP1*#8-3, *AVP1_*pro*_::IPP1*#17-3, *ICL_*pro*_::IPP1*#9-2, *ICL_*pro*_::IPP1*#11-2, and *ICL_*pro*_::IPP1*#24-1 lines. **(D)** Hierarchical clustering heat map of the relative abundances of metabolites. Scale indicates the color code relative to normalized metabolite abundance.

The CE-TOF MS analysis detected 136 metabolites (Supplementary Table 1), of which 115 were significantly altered (Supplementary Table 2). In *fugu5-1*, 52 and 5 metabolites were significantly increased and decreased, respectively, when compared to the WT (Supplementary Table 2). To gain insight into the metabolic changes correlated with tissue-specific removal of PPi, we performed principal component analysis (PCA) on the WT, *fugu5-1*, *AVP1_*pro*_::IPP1*#8-3, *PDF1_*pro*_::IPP1*#6-7, *PDF1_*pro*_::IPP1*#9-1, *CLV1_*pro*_::IPP1*#8-3, and *CLV1_*pro*_::IPP1*#12-3; and *PDF1_*pro*_::IPP1*#6-7^(+/–)^ and *CLV1_*pro*_::IPP1*#8-3^(+/–)^ lines. The first (PC1) and second (PC2) components contributed 50.7% of the total variance (Figure 6A). A scores plot indicated that *fugu5-1 CLV1_*pro*_::IPP1* was separable from the WT, *AVP1_*pro*_::IPP1*, *PDF1_*pro*_::IPP1*, and *fugu5-1*, yet *CLV1_*pro*_::IPP1* lines and *fugu5-1* were closely aligned on the PC1 axis (Figure 6A). The *PDF1_*pro*_::IPP1* and *PDF1_*pro*_::IPP1*#6-7^(+/–)^ and *CLV1_*pro*_::IPP1*#8-3^(+/–)^ were closer to the WT and *AVP1_*pro*_::IPP1* (Figure 6A). In addition, a biplot of the PCA scores (Supplementary Figure 7A) showed the metabolites’ contributions to PC1 and PC2 and identified 35 and 23 metabolites positively or negatively correlated with the PC1 and PC2 scores, respectively (Supplementary Figure 7B). Based on the factor loading scores, sugars, sugar phosphates, sugar amino acids, sugar nucleotides, and amino acids (Val, Ile, and Met) were significantly positively correlated with PC1 scores. By contrast, polyamines were negatively correlated with PC1.

The changes in the metabolic profile across the genotypes were recapitulated by visualization of the relative abundances of metabolites by means of Hierarchical Clustering Analysis (HCA) (Figure 6B). More specifically, HCA identified 10 clusters in total, among which metabolites that exhibited a positive correlation along PC1 axis were enriched in clusters 5 and 8 (Figures 6A,B and Supplementary Figure 7). On the contrary, metabolites that exhibited a positive correlation along PC2 axis were enriched in cluster 6 (Figures 6A,B and Supplementary Figure 7).

### Effect of the removal of inorganic pyrophosphate (PPi) immediately after seed imbibition on the metabolic profile of *ICL_*pro*_*::*IPP1*

Given that the expression of *ICL_*pro*_::IPP1* on the *fugu5-1* background rescued developmental defects in palisade tissue, we assessed its effect on the metabolic profile immediately after seed germination.

The CE-TOF MS analysis detected 113 metabolites (Supplementary Table 3), of which 86 were significantly changed (Supplementary Table 4). The PCA of the metabolomics data showed that the global metabolic profile of *ICL_*pro*_::IPP1* was similar to the WT and different from *fugu5-1* (Figure 6C). Furthermore, a biplot of the PCA scores (Supplementary Figure 8A) showed the metabolites’ contributions to PC1 and PC2 and identified 22 and 12 metabolites positively or negatively correlated with the PC1 and PC2 scores, respectively (Supplementary Figure 8B). The factor loading scores indicated that the metabolites significantly positively correlated with PC1 scores in *ICL_*pro*_::IPP1* overlapped with those in *PDF1_*pro*_::IPP1* and *CLV1_*pro*_::IPP1*. Finally, a HCA heat map of metabolite abundance confirmed the metabolic similarities between the WT and *ICL_*pro*_::IPP1* (Figure 6D), whereby metabolites that exhibited a positive correlation along PC1 and PC2 axes were enriched in cluster 6 (Figures 6C,D; Supplementary Figure 8).

### *fugu5* central metabolism remarkably recover in *PDF1_*pro*_::IPP1* and *ICL_*pro*_::IPP1*

**FIGURE 7:**
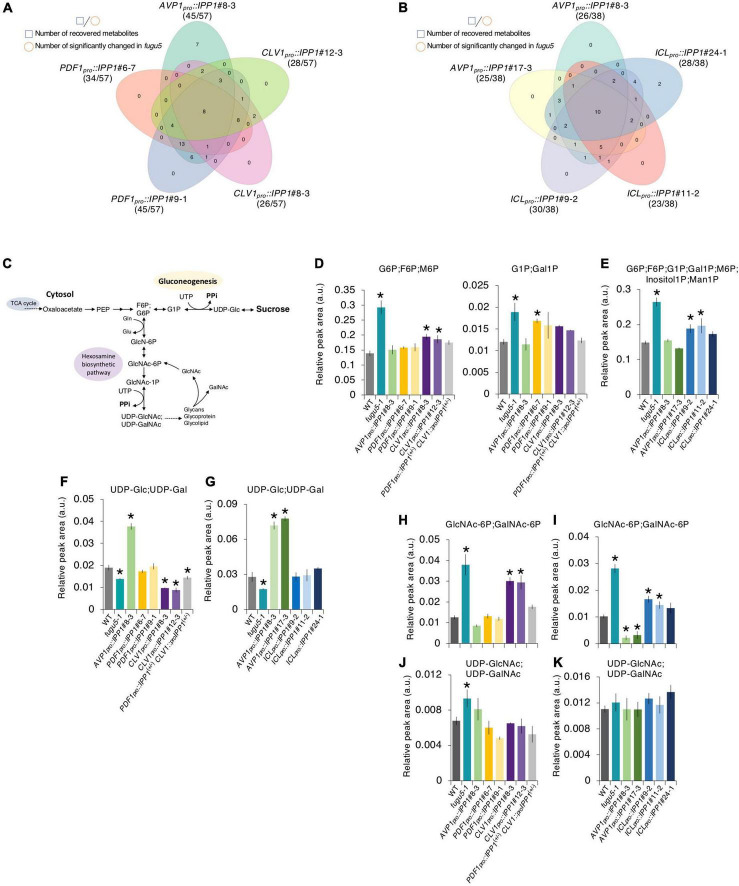
Effect on the metabolite profile of spatiotemporal expression of IPP1 on the *fugu5* background by capillary electrophoresis time-of-flight mass spectrometry (CE-TOF MS). **(A,B)** Venn diagram of metabolites that recovered to wildtype (WT) levels and those that exhibited opposite and significant fluctuations in *fugu5* vs. the indicated transgenic lines. **(C)** Schematic of the gluconeogenesis pathway targeted by inorganic pyrophosphate (PPi) overaccumulation in *fugu5-1* and the hexosamine biosynthetic pathway. **(D–K)** Fluctuations in major key metabolites in the indicated genotypes. Data are means ± SD (**E**, **F**, **H**, and **J:** *n* = 3; **E**, **G**, **I**, and **K:** *n* = 4). *Significant difference at *p* < 0.05 compared to the WT (Tukey’s HSD test).

We summarized the number of metabolites whose accumulation was significantly changed on the *fugu5-1* background and recovered to the WT level by tissue-specific expression of *IPP1* on the *fugu5-1* background (Figures 7A,B and Supplementary Tables 5, 6). *AVP1_*pro*_::IPP1* expression had the highest recovery rate (∼79%), followed by *PDF1_*pro*_::IPP1* and *CLV1_*pro*_::IPP1* (70 and 48%, respectively), in two independent transgenic lines (Figure 7A). Although 18 metabolites were commonly affected in *AVP1_*pro*_::IPP1* and *PDF1_*pro*_::IPP1*, only 5 showed similar trends between *AVP1_*pro*_::IPP1* and *CLV1_*pro*_::IPP1* (Figure 7A). It is important to note that expression of *ICL_*pro*_::IPP1* on the *fugu5-1* background yielded a recovery rate of ∼71%, which is roughly comparable to the 67% recovery rate in *AVP1_*pro*_::IPP1* (Figure 7B).

Excess PPi *in planta* affected gluconeogenesis (Figure 7C) by inhibiting UDP-Glc production (Ferjani et al., 2018). The overaccumulation of glucose 6-phosphate (G6P), a precursor of UDP-Glc, in *fugu5-1* was largely recovered in *PDF1_*pro*_::IPP1*, *CLV1_*pro*_::IPP1*, and *ICL_*pro*_::IPP1* (Figures 7D,E). The levels of G1P and (GlcNAc-6P; GalNAc-6P), whose accumulation increased in *fugu5-1*, were reduced in these transgenic lines (Figures 7D,H,I). It should be noted, however, that (UDP-GlcNAc; UDP-GalNAc) levels, which were expected to decrease in the *fugu5-1* background due to excess PPi (Figure 7C), were either slightly increased or unchanged (Figures 7J,K). This counter-intuitive result may indicate that the metabolic reaction converting GlcNAc-1P into (UDP-GlcNAc; UDP-GalNAc) is less susceptible to PPi-inhibition *in vivo*. For instance, PPi has relatively little effect on Arabidopsis PPDK activity *in vitro* (Chastain et al., 2011). Finally, although UDP-Glc decreased in *fugu5-1*, it was comparable to the WT in *PDF1_*pro*_::IPP1* and *ICL_*pro*_::IPP1* (Figures 7F,G). This rescue of UDP-Glc was less in *CLV1_*pro*_::IPP1*, likely because subepidermal mesophyll cell-specific removal of PPi had little impact on metabolism (Figures 6A, 7A).

## Discussion

The concentration of PPi *in planta* is under the tight control of several PPases that make different contributions to PPi homeostasis and cooperate to prevent its accumulation to a toxic level (Ferjani et al., 2011, 2018; Segami et al., 2018). Proliferating cells generate large amounts of PPi owing to their active metabolism, which correlates with their high expression of PPases (Segami et al., 2018, and references therein). Excess PPi triggers a plethora of growth and developmental defects, but little is known about its effect on major growth stages and/or plant tissue.

Whereas most *fugu5* phenotypes, such as CCE and hypocotyl elongation defects, are attributable to reduced production of Suc from TAG (Ferjani et al., 2011), PC morphological defects are likely triggered by excess PPi (Gunji et al., 2020). Although phenotypic and functional analyses indicate different mechanisms for the palisade and the epidermal developmental defects, the possibility that both are triggered by PPi-related metabolic disorders cannot be ruled out. Hence, the CCE-Suc and PCs-PPi relationships suggest major differences in metabolic activity in the two leaf tissues. Therefore, the effects of excess PPi on metabolism in these tissues and its impact on organogenesis in plants, which is the major focus of this research, has been awaiting direct investigation.

### Effect of excess inorganic pyrophosphate (PPi) on cotyledon development

Manipulating the PPi level in a plant tissue and/or cell type is technically challenging (Ferjani et al., 2011, 2014a,b; Hanchi et al., 2018; Segami et al., 2018) because of its broad contribution to metabolism. However, the genetic approach adopted in this study overcame these technical difficulties.

Our results revealed that the inhibitory effects of excess PPi were canceled in *IPP1*-expressing tissue only (Figures 1, 2). Furthermore, the removal of excess PPi at early developmental stages was crucial for proper plant development (Figures 4, 5). It is interesting that the inhibitory effects of PPi depended on the developmental stage of leaf tissue cells. Because hydrolysis of PPi by *ICL_*pro*_::IPP1* abolished CCE, the intracellular metabolic state at early developmental stages resulting from excess PPi is likely the main trigger of CCE. However, PC developmental defects in *ICL_*pro*_::IPP1* were only partially restored, which indicates that PC morphogenesis is mediated by a PPi-related mechanism that is likely different, at least in part, from post-germinative metabolism.

In this study, we constructed chimeric plants with respect to their ability to hydrolyze PPi by restricting IPP1 expression spatially or temporally using tissue- or developmental stage-specific promoters. Hence, PPi hydrolysis in a given tissue would reveal its impact on plant morphogenesis and metabolism. This would enable comparative analyses based on altered metabolic profiles and the identification of key metabolites in the CCE-Suc and PCs-PPi relationships.

Consistent with our previous report (Ferjani et al., 2018), the levels of gluconeogenesis-related metabolites and amino acids (e.g., Ile, Val, Met, and GABA) were altered in *fugu5* because of excess PPi (Figure 6). In addition, the levels of these metabolites were restored by PPi hydrolysis in the epidermis (Figure 6). By contrast, PPi hydrolysis by *CLV1_*pro*_::IPP1* did not contribute to skotomorphogenesis because *CLV1_*pro*_::IPP1* showed PPi-specific metabolic changes and the Suc level was as low as in *fugu5* (Figures 3, 7). In fact, although *CLV1_*pro*_::GUS* expression was confined to the single paradermal layer of the cotyledonary palisade tissue, it was patchy and lower compared to *PDF1_*pro*_::GUS* (discussed further below). Hence, the partial repression of CCE in *CLV1_*pro*_::IPP1* may reflect an amalgam of cellular behavior (i.e., CCE was solely repressed in *IPP1*-expressing cells in a tissue-autonomous manner). Such biased cellular behavior is likely supported by metabolic products synthesized after germination in tissue from which PPi has been removed.

### Impact of excess inorganic pyrophosphate (PPi) on pavement cell (PC) morphogenesis

The PC formation has been proposed to involve local elongation of lobes, local restriction of dimples, or a combination of both (Fu et al., 2002, 2005, 2009; Xu et al., 2010; Zhang et al., 2011; Abley et al., 2013; Lin et al., 2013; Sampathkumar et al., 2014; Armour et al., 2015; Higaki et al., 2016; Majda et al., 2017; Sapala et al., 2018). In addition, although Roh of Plants (ROP) signaling and phytohormonal regulation of PC interdigitation play central roles in the determination of plant cell shape, the mechanical effects of the PC wall, and the mechanical interactions between the adjacent cells may be involved (Sampathkumar et al., 2014; Cosgrove, 2018; Sapala et al., 2018; Bidhendi et al., 2019; Cosgrove and Anderson, 2020; Haas et al., 2020; Liu et al., 2021).

Unlike palisade tissue cells, PCs lose their shape complexity because of excess PPi rather than compromised gluconeogenesis (Gunji et al., 2020). This is in agreement with the failure of exogenous Suc to restore PC shapes in the *fugu5* mutant and with the ability of *icl-2*, *mls-2*, and *pck1-2* mutants (which have defects in TAG-to-Suc conversion) to form typical jigsaw puzzle–shaped PCs (Gunji et al., 2020). Furthermore, PPi removal in *AVP1_*pro*_::IPP1*, which ubiquitously expresses the yeast sPPase IPP1, restores PC developmental defects (Gunji et al., 2020). Consistently, exclusive expression of IPP1 in the epidermis in *PDF1_*pro*_::IPP1* confirms this hypothesis and suggests that PPi has its inhibitory effects *in planta* in a tissue- and cell-autonomous manner.

Despite the fact that IPP1 expression in *PDF1_*pro*_::IPP1* was confined to the outermost layer, cell number and size in palisade tissue recovered partially compared to the *fugu5* single mutant (Figure 2). IPP1 transport from its target layer to neighboring layers is unlikely to underlie partial recovery of the compensation phenotype in transgenic lines expressing IPP1 in a tissue-specific manner. How can this discrepancy be explained?

First, if we assume that TAG in PCs of *PDF1_*pro*_::IPP1* is fully converted into Suc, and provided that Suc is highly mobile, Suc may translocate from the epidermis to the palisade tissue, reactivating cell cycling and thereby inhibiting CCE. This partial recovery appears to be plausible provided that the *de novo* synthesis of Suc from TAG in *PDF1_*pro*_::IPP1* is inferior to that in *AVP1_*pro*_::IPP1*, because the former is epidermis specific and the latter is ubiquitously expressed. This is confirmed by the significantly lower Suc content in *PDF1_*pro*_::IPP1* compared to *AVP1_*pro*_::IPP1*#8-3 (Figure 3).

Second, upon the expression of *PDF1_*pro*_::IPP1* on the *fugu5-1* background, a relaxed epidermis may allow proliferation and growth of mesophyll and other inner tissues in cotyledons. In this case, total or partial relief of the mechanical constraints exerted by the epidermis, the load-bearing layer (Asaoka et al., 2021), toward the inner tissues may stimulate their growth, resulting in partial recovery of their cellular phenotypes. Indeed, this may explain the recovered hypocotyl elongation in *PDF1_*pro*_::IPP1* (Figures 3A,B).

Arabidopsis cotyledons are polarized with an adaxial side containing the palisade tissue and abaxial side containing the spongy parenchyma, all of them being wrapped by the single-layered epidermis on both sides. This has mechanical implications (e.g., see Onoda et al., 2015). More generally, the plant cells are enclosed by stiff cell walls, and the associated mechanical properties and signals should be integrated with genetic networks to fully understand their development (Autran et al., 2021; Trinh et al., 2021; Nakayama et al., 2022). Unlike cylindrical flowering stems with a relatively limited flexibility in growth direction, flat leaves where the stress patterns are relatively isotropic can grow with a higher degree of freedom (Zhang et al., 2011; Verger et al., 2018). Nonetheless, the computational modeling of stress patterns suggested that leaves are experiencing strongly anisotropic tensile stress across the organ thickness (Zhao et al., 2020), that if combined with CCE might further yield to stress. Importantly, excess PPi has recently been reported to cause morphological changes and weakening of cell walls (Segami et al., 2018), to reduce the polymerization velocity of cortical MTs, which appeared slightly fragmented and sparse (Gunji et al., 2020). Consistently, PPi delayed tubulin polymerization *in vitro* in a dose-dependent manner, thus supporting a link between PPi and MT dynamics (Gunji et al., 2020). Taking this into account, it seems that perturbation of cell wall stiffness and/or mechanosensing could also be amongst the most relevant factors, in addition to the above two possibilities, to explain the effect of excess PPi on PC growth and patterning.

### Impact of excess inorganic pyrophosphate (PPi) on compensated cell enlargement (CCE)

Compensated cell enlargement in *CLV1_*pro*_::IPP1* was not completely suppressed—the cell number and size of palisade tissue were only partially restored. Although the subepidermal cell-specific expression of *IPP1* against *fugu5-1* significantly altered the accumulation of key metabolites, including G6P, its impact was limited compared to the other transgenic lines (Figures 3, 6, 7). For instance, the decreased level of Suc in *fugu5-1* was rescued in *PDF1_*pro*_::IPP1* and *ICL_*pro*_::IPP1* but not in *CLV1_*pro*_::IPP1*. These results are consistent with the fact that CCE induction is triggered by a decrease in cell number due to a reduced Suc content in *fugu5-1* (Tabeta et al., 2021).

The GUS staining of *CLV1_*pro*_::GUS* showed low and patchy *CLV1* promoter activity; by contrast, the *PDF1* promoter was strongly and uniformly expressed. This suggests that PPi is less efficiently removed in the target tissue of *CLV1_*pro*_::IPP1* compared to *PDF1_*pro*_::IPP1*. In addition, the expression pattern may explain the discrepancy among the phenotypes of palisade tissue cells of the *CLV1_*pro*_::IPP1*#8-3, #10-2, and #12-3 lines.

In one of our experiments, we checked whether PPi removal in the double transgenic heterozygous lines would contribute to suppressing CCE. Interestingly, while our data revealed that CCE was totally suppressed in *PDF1_*pro*_::IPP1*^(+/–)^ *CLV1_*pro*_::IPP1*^(+/–)^ (Supplementary Figure 4), at the same time it raised the following question: Why did *PDF1_*pro*_::IPP1* expression alone fail to restore normal cell division in palisade tissue cells, particularly when considering the highly mobile nature of sucrose? This question is open for future investigation to identify the critical factors behind this intriguing phenotype.

### Inorganic pyrophosphate (PPi) removal from leaf tissue at different developmental stages reveals different cellular needs for proper morphogenesis

Furthermore, PPi homeostasis has been investigated in *fugu5* mutants (Ferjani et al., 2011; Kriegel et al., 2015; Asaoka et al., 2016, 2019; Fukuda et al., 2016; Wang et al., 2016; Segami et al., 2018; Gunji et al., 2020; Tabeta et al., 2021). The H^+^-PPase is crucial for plant development, in particular, during the mobilization of seed nutrient reserves, not as a proton pump but rather as a PPi-hydrolyzing enzyme (Ferjani et al., 2014a; Segami et al., 2018). It is intriguing that PPi accumulation may affect different leaf tissues or cell types by different mechanisms. For instance, the effects of a lack of Suc or its exogenous supply raise questions about the implication of PPi in any metabolic activity in the epidermis.

The reactivation of post-germinative cell division relies exclusively on energy produced by TAG mobilization (Bewley and Black, 1994; Graham, 2008). Nonetheless, whereas compensation was abolished, PC morphology did not fully recover in *ICL_*pro*_::IPP1*. In *ICL_*pro*_::IPP1*, PPi was hydrolyzed efficiently by the transgene, because cell number and size recovered to WT levels. This is in agreement with the fact that the *icl-2* mutant displays compensation and is classified with *mls-2* and *pck1-2* as showing class II CCE (Takahashi et al., 2017). Whereas PC morphology in all class II mutants was normal, *fugu5* had simpler PCs than the WT (Gunji et al., 2020). There could be many reasons for this. First, TAG-to-Suc conversion is immediately necessary for reactivation of cell division in palisade tissue after seed imbibition. Second, the lack of Suc is the main reason for class II CCE. Third, excess PPi on the *fugu5* background inhibits gluconeogenesis, but in *icl-2*, *mls-2*, and *pck1-2* the PPi level is canceled by their functional H^+^-PPases. Finally, PCs in *icl-2*, *mls-2*, and *pck1-2* are normally shaped. This indicates that excess PPi, not lack of Suc, is related to PC development.

Taken together, these findings demonstrate that palisade tissue requires FUGU5 for a few days after seed imbibition, but prolonged H^+^-PPase activity is required for PCs to adopt their typical jigsaw puzzle shape (Higaki et al., 2016). In other words, the decision to trigger CCE in cotyledons occurs early during postembryonic development and is followed by large-scale metabolic reprogramming involving IBA-to-IAA conversion as a key step in CCE induction (Tabeta et al., 2021). By contrast, PCs require more time than the palisade to mature and gradually adopt complex shapes (Fu et al., 2002, 2005; Armour et al., 2015; Higaki et al., 2016). Hence, the partial recovery in *ICL_*pro*_::IPP1* indicates that PPi homeostasis must be maintained for a long period for the UI value to reach that of the WT. The complete recovery of palisade tissue cells and the partial recovery of PCs in the epidermis indicates that although PPi homeostasis is necessary for both tissues, leaf tissues have different cellular needs for proper morphogenesis.

## Conclusions and future prospects

The H^+^-PPase regulates both vacuolar acidification and cytosolic PPi concentration in living plant cells. Although various mechanisms have been proposed to explain its role in plant growth and development, a role in PPi homeostasis has been suggested. Hence, although the holistic contribution of H^+^-PPase to plant growth has been investigated, its actions in different tissues and cell types throughout the plant life cycle with and without abiotic stresses are unclear (Schilling et al., 2017). This study provides a conceptual overview of how these questions should be approached. No direct measurement of PPi levels in major leaf tissues and/or cell types has been conducted because this is still technically challenging. Nonetheless, our previous works (Asaoka et al., 2019; Gunji et al., 2020; Tabeta et al., 2021), together with the spatiotemporal approach used in this study, unambiguously show that PPi has different effects on different plant tissues and cell types and at different developmental stages.

## Data availability statement

The original contributions presented in the study are included in the article/Supplementary material, further inquiries can be directed to the corresponding author.

## Author contributions

AF conceived the project, designed, supervised, and funded the study. SG performed the experiments, conducted the phenotyping analyses, and analyzed the data. SG, AF, and GH established the transgenic lines. KK and AO performed the CE-TOF MS metabolomic analyses. HTa performed Suc quantification. MA assisted metabolome data analyses. HTs and MH directed and funded the study. SG and AF wrote the manuscript with inputs from all co-authors. All authors read and approved the final manuscript.

## Footnotes

1
https://www.metaboanalyst.ca

## Abbreviations

H^+^-PPase: H^+^-translocating pyrophosphatase
sPPase: soluble inorganic pyrophosphatase
PPi: inorganic pyrophosphate
PFP: PPi-dependent phosphofructokinase
UGPase: UDP-glucose pyrophosphorylase
PPDK: pyruvate orthophosphate dikinase
DAS: days after seed sowing
DAI: days after induction of seed germination.
