# Supplementary material for: Tissue-targeted inorganic pyrophosphate hydrolysis in a fugu5 mutant reveals that excess inorganic pyrophosphate triggers developmental defects in a cell-autonomous manner
Source: Front Plant Sci. 2022 Aug 4;13:945225. doi: 10.3389/fpls.2022.945225 (PMC9386291; doi:10.3389/fpls.2022.945225)
Supplement: Supplementary file 1 [file Data_Sheet_1.pdf]

Tissue-targeted inorganic pyrophosphate hydrolysis in a *fugu5* mutant reveals that excess inorganic pyrophosphate triggers developmental defects in a cell-autonomous manner

Shizuka Gunji, Kensuke Kawade, Hiromitsu Tabeta, Gorou Horiguchi, Akira Oikawa, Mariko Asaoka, Masami Yokota Hirai, Hirokazu Tsukaya, Ali Ferjani

Supplementary Material

1 Supplementary Figures

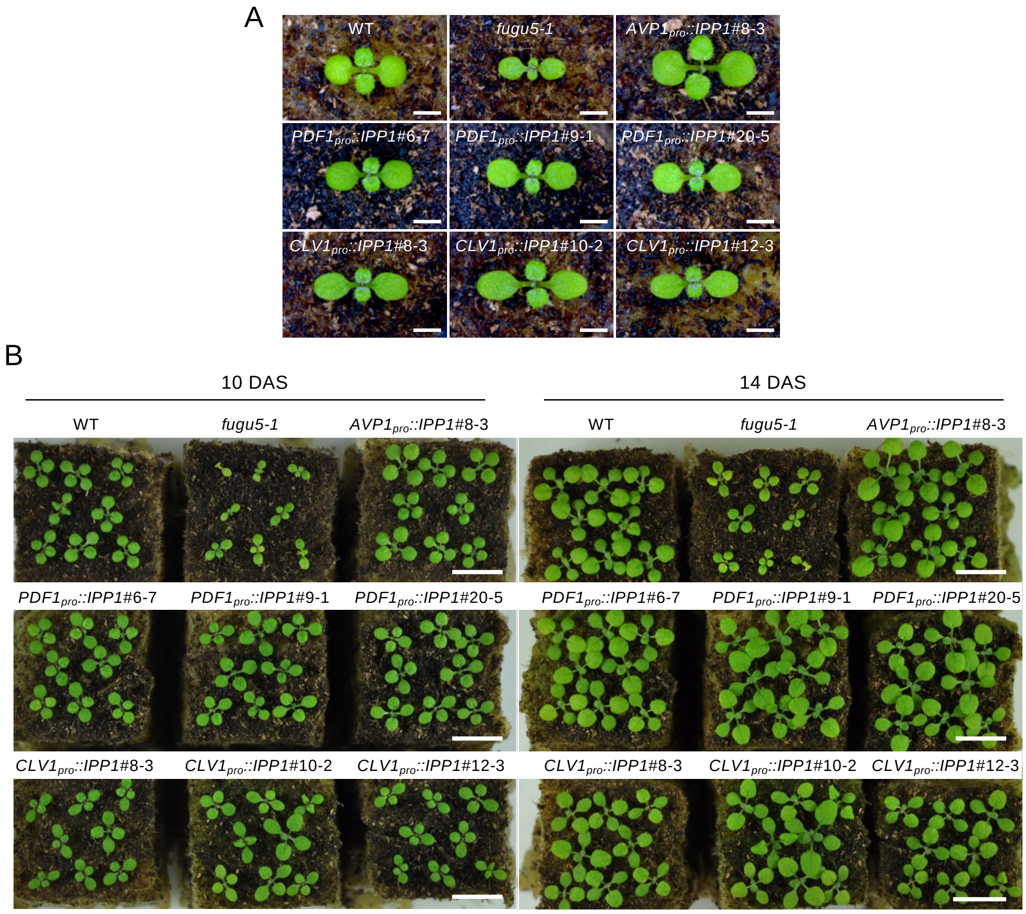

Figure S1. Gross phenotype of transgenic lines in which PPi was removed from the epidermis (*PDF1*$_{pro}$*::IPP1*) or palisade tissue cells (*CLV1*$_{pro}$*::IPP1*) in a tissue-specific manner.

(A) Gross morphology of the WT, *fugu5-1*, *AVP1*$_{pro}$*::IPP1*#8-3 (Ferjani *et al.*, 2011), *PDF1*$_{pro}$*::IPP1*#6-7, *PDF1*$_{pro}$*::IPP1*#9-1, *PDF1pro::IPP1*#20-5, *CLV1*$_{pro}$*::IPP1*#8-3, *CLV1*$_{pro}$*::IPP1*#10-2, and *CLV1*$_{pro}$*::IPP1*#12-3 (this study) plants grown on rockwool for 14 DAS. Scale bar = 2 mm.
(B) Photograph of each genotype grown on rockwool. Transgenic *IPP1* lines showed growth phenotype recovery compared to the *fugu5.* Scale bar = 1 cm. DAS, days after seed sowing.

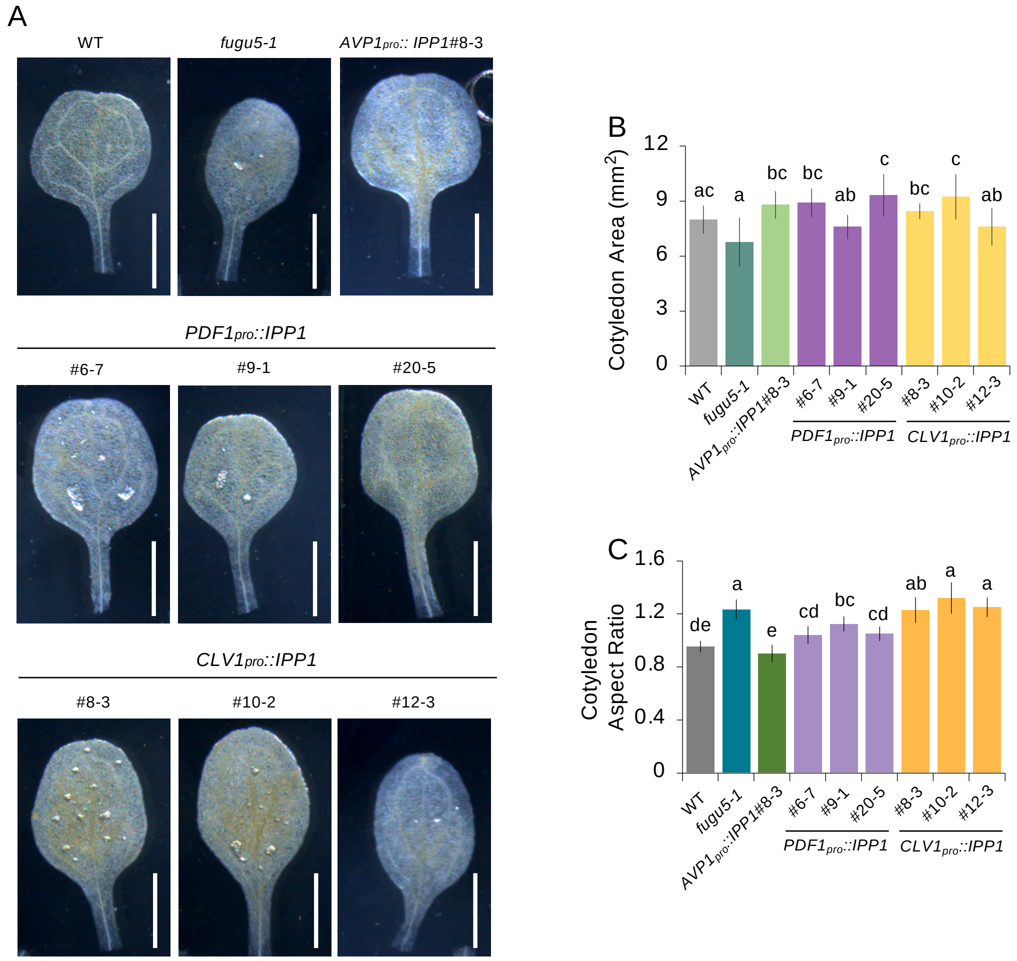

Figure S2. Cotyledon phenotype of transgenic lines in which PPi was removed in a tissue-specific manner.

(A) Micrographs of cleared cotyledons of the WT, *fugu5-1*, *AVP1*$_{pro}$*::IPP1*#8-3, *PDF1*$_{pro}$*::IPP1*#6-7, *PDF1*$_{pro}$*::IPP1*#9-1, *PDF1*$_{pro}$*::IPP1*#20-5, *CLV1*$_{pro}$*::IPP1*#8-3, *CLV1*$_{pro}$*::IPP1*#10-2, and *CLV1*$_{pro}$*::IPP1*#12-3 collected at 25 DAS. Scale bar = 2 mm.
(B) Cotyledon area of mature cotyledons at 25 DAS. Areas are means ± SD ($n \geq 9$ cotyledons). Each character represents a significant difference at $P < 0.05$ (Tukey's HSD test).
(C) Aspect ratio of the cotyledon at 25 DAS. Cotyledon aspect ratio was calculated by dividing cotyledon blade length by cotyledon blade width. Longer cotyledons have greater aspect ratio values. Each character represents a significant difference at $P < 0.05$ (Tukey's HSD test). DAS, days after seed sowing.

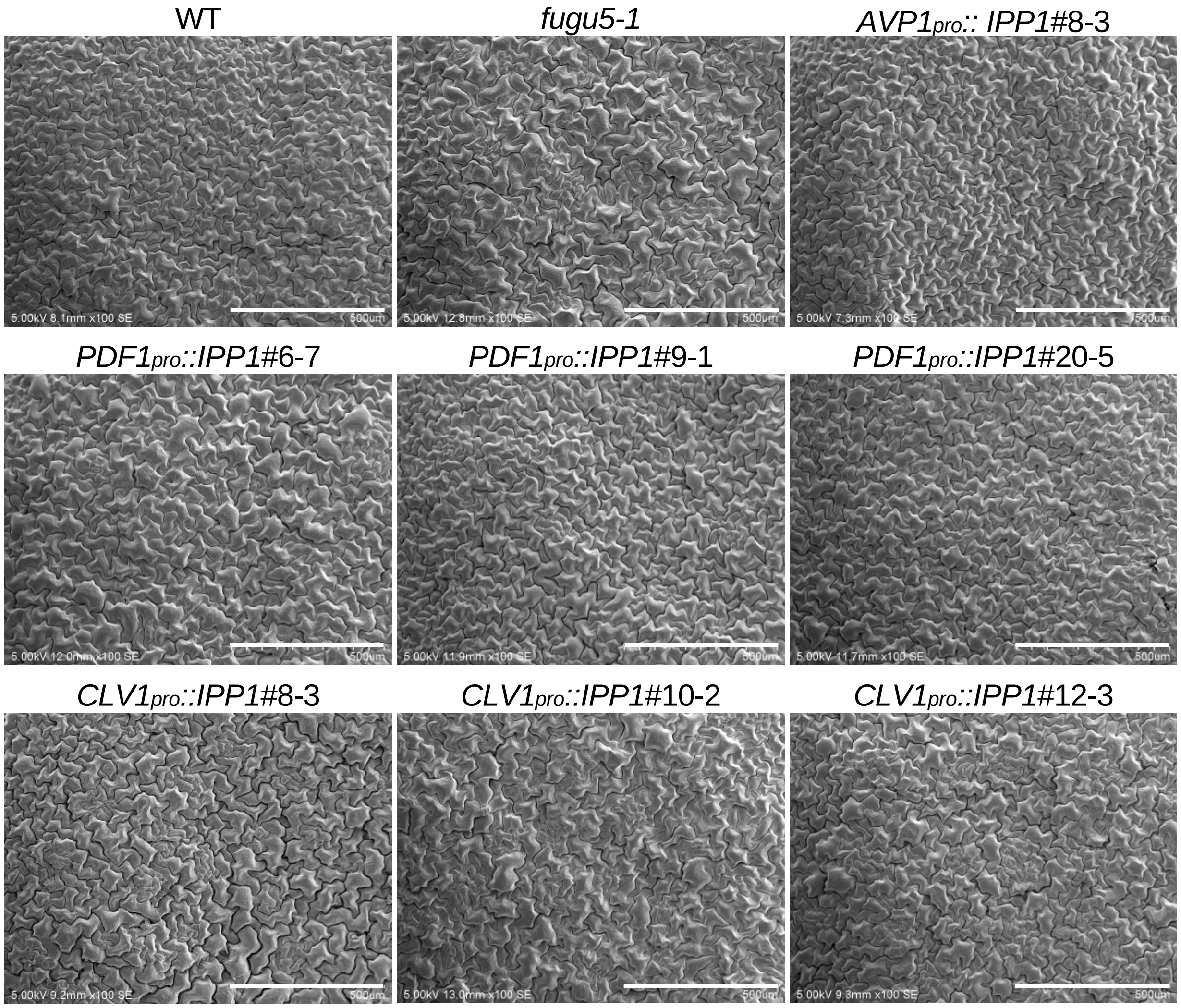

Figure S3. Scanning electron microscope (SEM) images of the adaxial side of cotyledons of transgenic plants expressing IPP1 in a tissue-specific manner.

SEM images showing the adaxial side of cotyledons of the WT, *fugu5-1*, *AVP1*$_{pro}$::*IPP1*#8-3, *PDF1*$_{pro}$::*IPP1*#6-7, *PDF1*$_{pro}$::*IPP1*#9-1, *PDF1*$_{pro}$::*IPP1*#20-5, *CLV1*$_{pro}$::*IPP1*#8-3, *CLV1*$_{pro}$::*IPP1*#10-2 and *CLV1*$_{pro}$::*IPP1*#12-3 at 25 DAS. Scale bar = 500 µm. Pavement cells and stomata within representative images have been traced and color-coded (See Figure 1F). DAS, days after seed sowing.

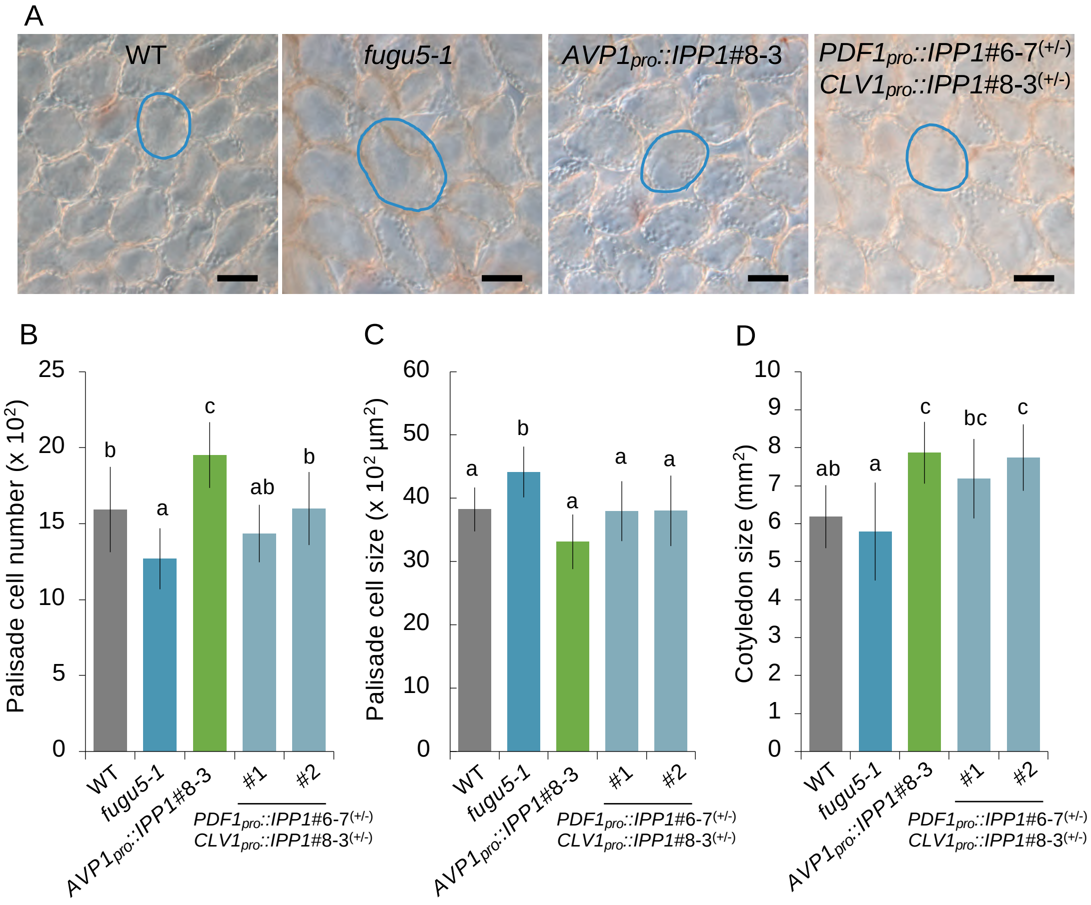

Figure S4. Palisade tissue cellular phenotype of the cotyledon of *PDF1$_{pro}$::IPP1$^{(+/-)}$ CLV1$_{pro}$::IPP1$^{(+/-)}$*.

(A) Micrographs of palisade tissue cells from cleared cotyledons of the WT, *fugu5-1*, *AVP1$_{pro}$::IPP1#8-3* and *PDF1$_{pro}$::IPP1*#6-7$^{(+/-)}$ *CLV1$_{pro}$IPP1*#8-3$^{(+/-)}$ lines at 25 DAS. Contours in blue were drawn to highlight representative cell size per each genotype. Scale bar = 50 µm.

(B-D) Number of subepidermal palisade tissue cells (B), and their average sizes (C) and cotyledon size (D). Data are means ± SD (*n* = 11 cotyledons) . Each character represents a significant difference at *P* < 0.05 (Tukey's HSD test) . DAS, days after seed sowing.

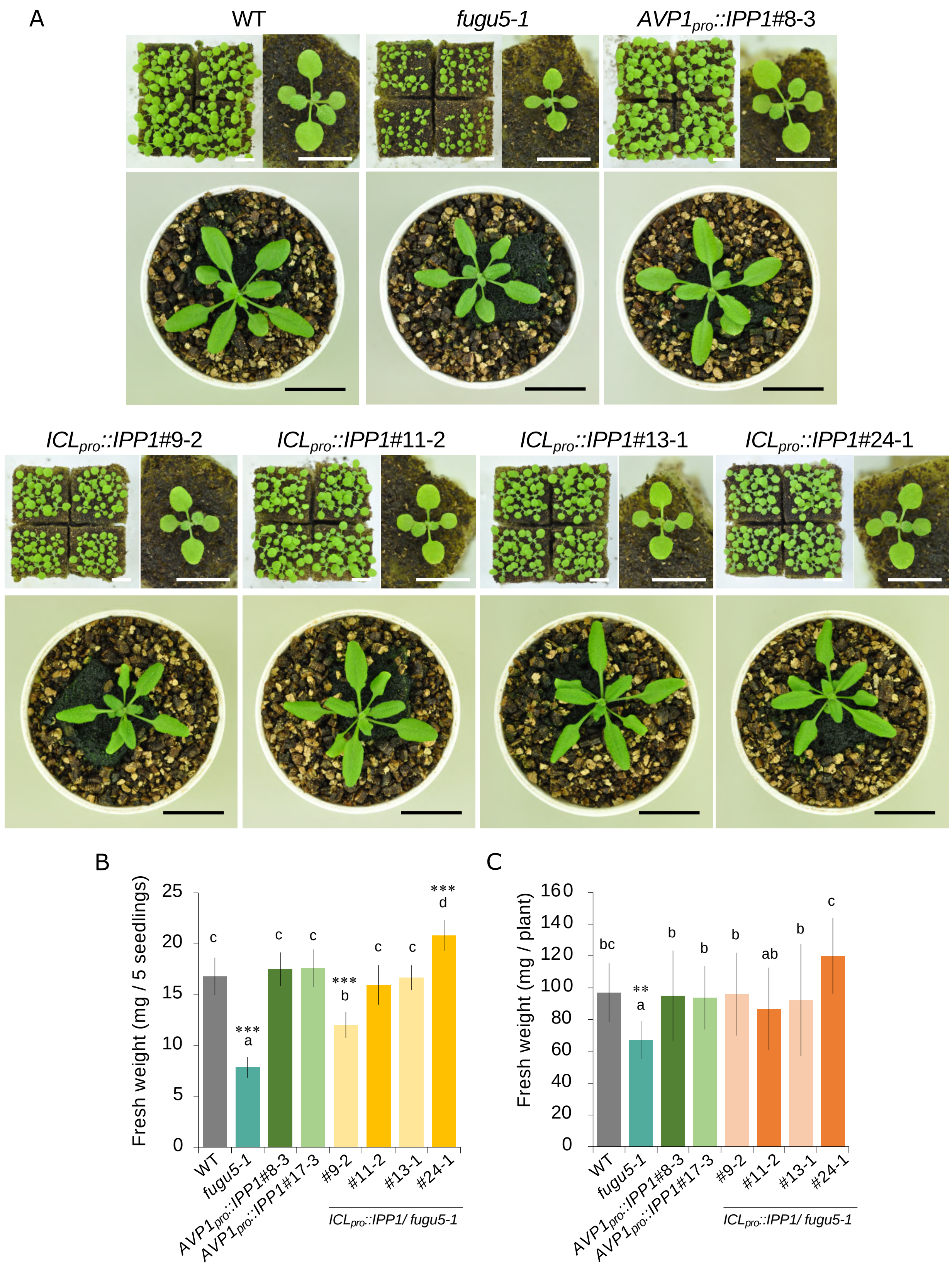

Figure S5. Growth phenotype and fresh weight of *ICL$_{pro}$::IPP1*.

(A) Gross morphology of representative transgenic lines grown on rockwool for 14 DAS (upper panels) and 23 DAS (lower panels). Scale bar = 1 cm (upper panels), 2 cm (lower panels). DAS, days after seed sowing.
(B-C) Fresh weight (FW) of the WT, *fugu5-1* and representative *ICL$_{pro}$::IPP1* transgenic lines. (B) FW of 12 DAS seedlings grown on rockwool (*n* = 10). (C) FW of 25 DAS plants (*n* = 20). Results are means ± SD. Each character represents a significant difference at $P < 0.05$ (Tukey's HSD test). **, Significant difference at $P < 0.01$, and ***, significant difference at $P < 0.0001$ compared to the WT.

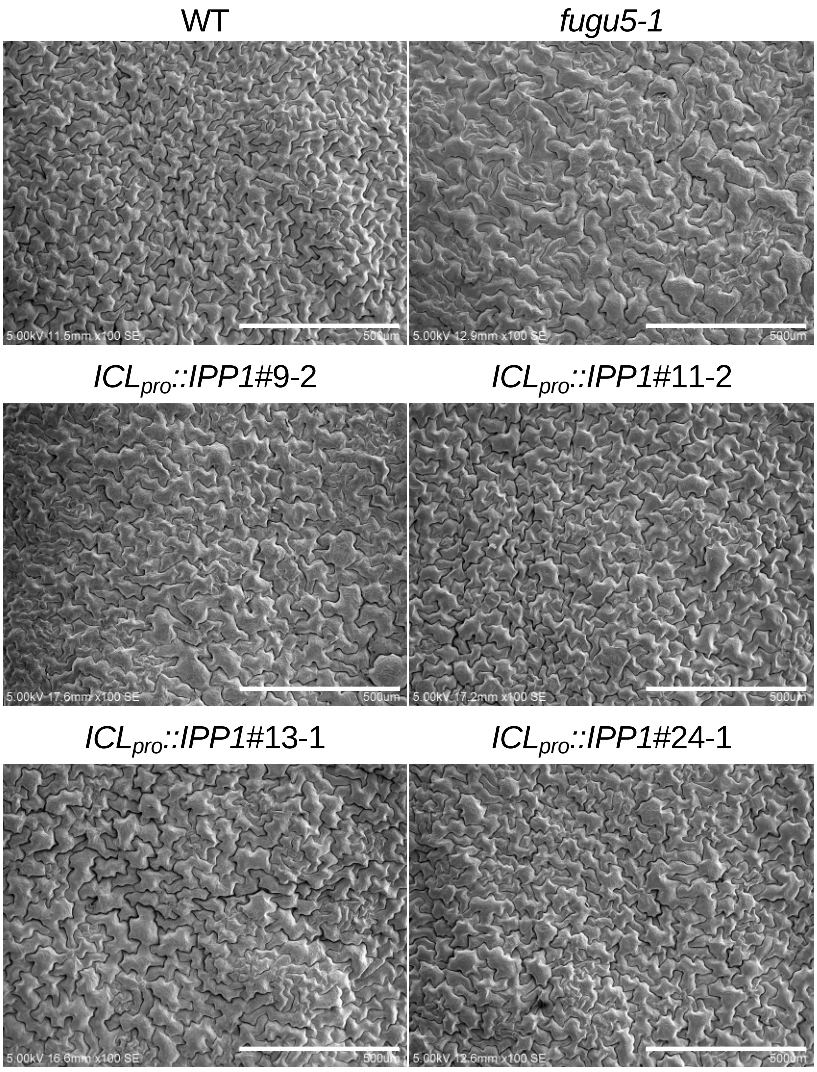

Figure S6. Scanning electron microscope (SEM) images of the adaxial side of cotyledons of transgenic plants expressing IPP1 in a developmental stage–specific manner.

SEM images of cotyledons adaxial side of the WT, *fugu5-1*, *ICL$_{pro}$::IPP1*#9-2, *ICL$_{pro}$::IPP1*#11-2, *ICL$_{pro}$::IPP1*#13-1, and *ICL$_{pro}$::IPP1*#24-1 at 25 DAS. Scale bar = 500 µm. Pavement cells and stomata within representative images have been traced and color-coded (See Figure 4E). DAS, days after seed sowing.

A

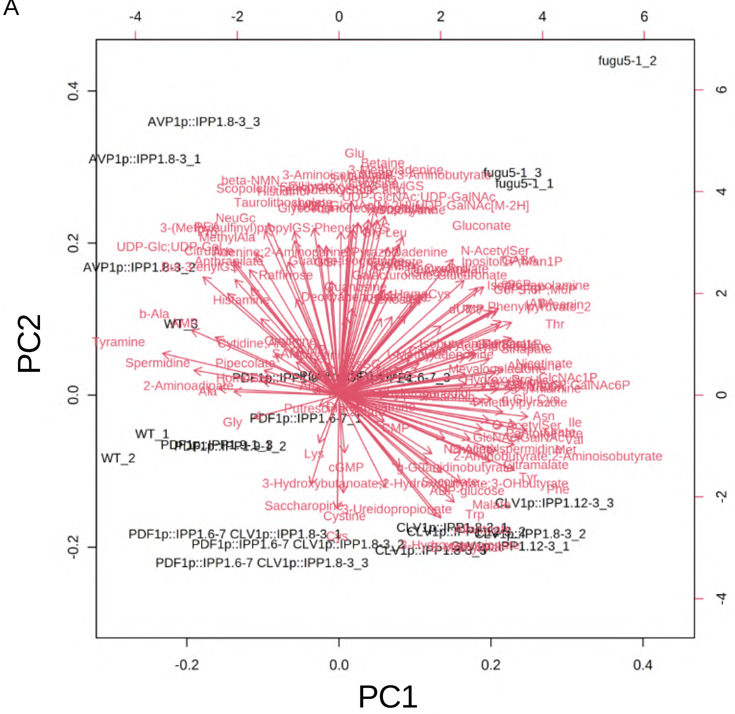

B

Metabolite	Factor loading (PC1)
Ile	0.94725874
Val	0.94244594
GlcNAc6P;GalNAc6P	0.92239258
GlcNAc1P	0.91899894
Met	0.90881801
Urate	0.88728379
Phe	0.87957096
2-Aminobutyrate;2-Aminoisobutyrate	0.87895393
Thiamine	0.86587298
Thr	0.86550276
L-Theanin	0.86531765
Pantothenate	0.82354497
Nicotinate	0.82280454
Asn	0.82212581
ABA	0.81447469
Phenylpyruvate_2	0.80349162
His	0.80256608
Isopropanolamine	0.80022138
g-gluBCA	0.79849371
Citramalate	0.79034896
G6P;F6P;M6P	0.78689361
Agmatine	0.78504254
g-Glu-Cys	0.76690196
S7P	0.76585302
Tyr	0.75943594
O-AcetylSer	0.75314228
Sinapate	0.75258695
N6-AcetylLys	0.73191657
GABA	0.72506758
S6P	0.72253777
GlcNAc;GalNAc	0.72111861
4-Methylpyrazole	0.72037818
Spermidine	-0.7270421
b-Ala	-0.7405549
Tyramine	-0.884754

Metabolite	Factor loading (PC2)
Glu	0.97161137
Betaine	0.93282185
3-Methyladenine	0.89904632
Gly3P	0.88058824
3-Aminoisobutyrate;3-Aminobutyrate	0.87910057
3-MethylHis	0.8647749
beta-NMN	0.86284644
Cytosine	0.84598622
Shikimate	0.83628884
2-Hydroxybut-3-enylGS	0.83402979
Scopoletin	0.82416712
GSSG	0.82323044
Taurodeoxycholic acid	0.81909803
Histidinol	0.81871234
UDP-GlcNAc;UDP-GalNAc	0.79320162
Taurolithocholate	0.77430275
UDP-GlcNAc[M-2H];UDP-GalNAc[M-2H]	0.75639565
NAD_1	0.75364071
Asp	0.74840633
Glycochenodeoxycholate	0.74642278
Theophylline	0.74184958
Stachydrine	0.73859876
NeuGc	0.71071879

Figure S7. CE-TOF MS analysis of the metabolic profiles of *PDF1$_{pro}$::IPP1* and *CLV1$_{pro}$::IPP1*.

(A) Biplot of PCA scores plot from Figure 6A shows each metabolite contribution. Each red arrowhead (length and direction) in the biplot indicates individual metabolite correlations represented by PC1 and PC2. Data are three independent experiments from WT, *fugu5-1*, *AVP1$_{pro}$::IPP1*#8-3, *PDF1$_{pro}$::IPP1*#6-7, *PDF1$_{pro}$::IPP1*#9-1, *CLV1$_{pro}$::IPP1*#8-3, *CLV1$_{pro}$::IPP1*#12-3 and *PDF1$_{pro}$::IPP1*#6-7$^{(+/-)}$ *CLV1$_{pro}$::IPP1*#8-3$^{(+/-)}$. Note that *AVP1$_{pro}$::IPP1*#8-3 has been abbreviated within the biplot as AVP1p::IPP1.8-3 for convenience. Same abbreviation has been applied for the other transgenic lines. The last number in each line name (i.e., _1; _2, and_3) indicates sample number.
(B) Factor loadings for PC1 and PC2 are listed. Absolute value of the factor loading is ≥ 0.7, indicating that there is a positive or a negative correlation between the PC scores and each metabolite.

A

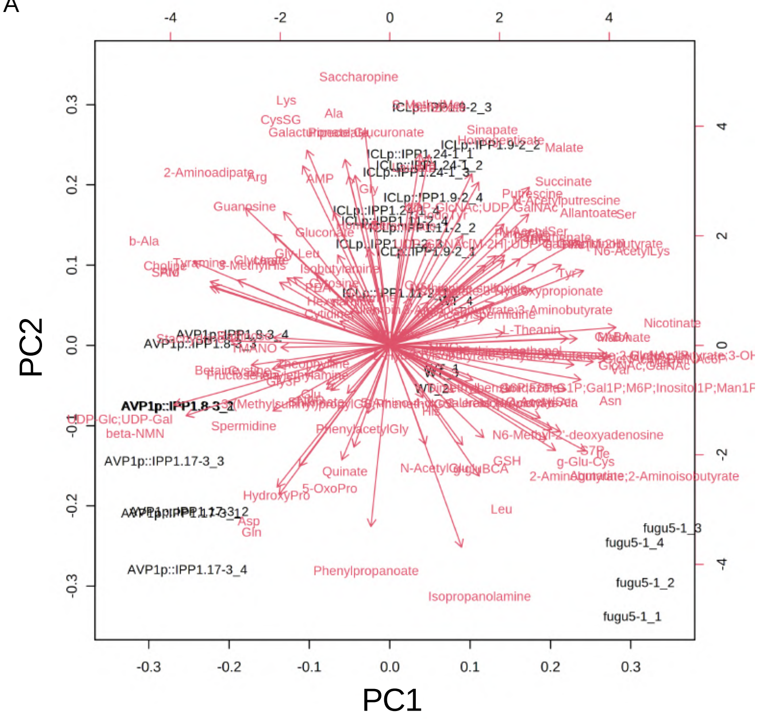

B

Metabolite	Factor loading (PC1)
Nicotinate	0.97488262
GlcNAc6P;GalNAc6P	0.94846553
Met	0.93202944
GlcNAc1P	0.92910024
GlcNAc;GalNAc	0.876917
2-Aminobutyrate;2-Aminoisobutyrate	0.84355662
N6-AcetylLys	0.83493174
G6P;F6P;G1P;Gal1P;M6P;Inositol1P;Man1P	0.82288946
Ser	0.81415609
Malonate	0.80699581
Val	0.79300072
GABA	0.7675058
Asn	0.76061674
g-Guanidinobutyrate	0.73870195
Ile	0.73566426
Agmatine	0.71456314
Pro	-0.7594234
Choline	-0.7734727
SAM	-0.7739066
b-Ala	-0.8465943
beta-NMN	-0.8777849
UDP-Glc;UDP-Gal	-0.9308903

Metabolite	Factor loading (PC2)
Homogentisate	0.92327136
GABA	0.84175119
Gly	0.82673547
Gln	0.82155613
Nicotinate	0.8017963
Benzoate	0.77437628
Arg	0.74025358
Cytidine	0.73350739
N8-Acetylspermidine	0.72998196
Lys	0.70399822
Tyramine	-0.7814271
N-AcetylGlu	-0.8707816

Figure S8. CE-TOF MS analysis of the metabolic profile of *ICL$_{pro}$::IPP1*.

(A) Biplot of PCA scores plot from Figure 6C shows each metabolite contribution. Each red arrowhead (length and direction) in the biplot indicates individual metabolite correlations represented by PC1 and PC2. Data are four independent experiments from WT, *fugu5-1*, *AVP1$_{pro}$::IPP1*#8-3, *AVP1$_{pro}$::IPP1*#17-3, *ICL$_{pro}$::IPP1*#9-2, *ICL$_{pro}$::IPP1*#11-2, *ICL$_{pro}$::IPP1*#24-1. Note that *AVP1$_{pro}$::IPP1*#8-3 has been abbreviated within the biplot as AVP1p::IPP1.8-3 for convenience. Same abbreviation has been applied for the other transgenic lines. The last number in each line name (i.e., _1; _2, _3, and _4) indicates sample number.
(B) Factor loadings for PC1 and PC2 are listed. Absolute value of the factor loading is ≥ 0.7, indicating that there is a positive or a negative correlation between the PC scores and each metabolite.
